# A gene regulatory network underlying the formation of pre-placodal ectoderm in *Xenopus laevis*

**DOI:** 10.1186/s12915-018-0540-5

**Published:** 2018-07-16

**Authors:** Santosh Kumar Maharana, Gerhard Schlosser

**Affiliations:** 1 School of Natural Sciences and Regenerative Medicine Institute (REMEDI), National University of Ireland, Biomedical Sciences Building, Newcastle Road, Galway, Ireland; 2 Present address: Department of Basic Science and Craniofacial Biology, College of Dentistry, New York University, New York City, NY USA

## Abstract

**Background:**

The neural plate border ectoderm gives rise to key developmental structures during embryogenesis, including the neural crest and the preplacodal ectoderm. Many sensory organs and ganglia of vertebrates develop from cranial placodes, which themselves arise from preplacodal ectoderm, defined by expression of transcription factor Six1 and its coactivator Eya1. Here we elucidate the gene regulatory network underlying the specification of the preplacodal ectoderm in *Xenopus*, and the functional interactions among transcription factors that give rise to this structure.

**Results:**

To elucidate the gene regulatory network upstream of preplacodal ectoderm formation, we use gain- and loss-of-function studies to explore the role of early ectodermal transcription factors for establishing the preplacodal ectoderm and adjacent ectodermal territories, and the role of Six1 and Eya1 in feedback regulation of these transcription factors. Our findings suggest that transcription factors with expression restricted to ventral (non-neural) ectoderm (AP2, Msx1, FoxI1, Vent2, Dlx3, GATA2) and those restricted to dorsal (neural) ectoderm (Pax3, Hairy2b, Zic1) are required for specification of both preplacodal ectoderm and neural crest in a context-dependent fashion and are cross-regulated by Eya1 and Six1.

**Conclusion:**

These findings allow us to elucidate a detailed gene regulatory network at the neural plate border upstream of preplacodal ectoderm formation based on functional interactions between ectodermal transcription factors. We propose a new model to explain the formation of immediately juxtaposed preplacodal ectoderm and neural crest territories at the neural plate border, uniting previous models.

**Electronic supplementary material:**

The online version of this article (10.1186/s12915-018-0540-5) contains supplementary material, which is available to authorized users.

## Background

The evolutionary success of vertebrates is largely due to the invention of a novel skull and new cranial sense organs and ganglia that allowed the adoption of a more active lifestyle. Many of these novel structures are derived from two embryonic tissues, the neural crest (NC) and the cranial placodes, which originated in vertebrate ancestors [1]. Whereas the NC contributes to the skull and forms pigment cells, glial cells and sensory neurons of the peripheral nervous system, cranial placodes form most of the paired sensory organs and contribute sensory neurons to the cranial ganglia. During embryogenesis, NC and cranial placodes arise from ectoderm located between the neural plate on the dorsal side and the epidermis on the ventral side, the so-called neural plate border (NPB) region, with NC originating from the lateral neural folds and cranial placodes from the pre-placodal ectoderm (PPE), a horseshoe-shaped region surrounding the anterior neural plate and anterior NC [2–4].

In the last two decades, substantial progress has been made in unravelling the gene regulatory network (GRN) underlying NC specification, but much less is known about the specification of the PPE. In a first step, the joint expression of a group of transcription factors (TFs) including Dlx3/5, AP2, Msx1, Zic1 and Pax3—designated as “NPB specifiers”—defines a relatively broad NPB region. These TFs then cooperate with BMP, Wnt and FGF signaling pathways to upregulate a second group of TFs including FoxD3, Snail1/2 and Sox9/10—the “NC specifiers”—in a more confined territory [5–7]. The latter cross-regulate each other and activate NC-specific effector genes, thereby specifying the proper NC [7].

While the interactions between NPB specifiers, NC specifiers and other TFs at the NPB appear to be overall conserved between different vertebrates, there are subtle differences in their spatial and temporal pattern of expression between species [4, 8, 9]. Because at any given time the spatial pattern of TF expression determines their regulatory relationships, which in turn determine the changes in their expression pattern over time, elucidation of functional regulatory interactions requires close consideration of spatiotemporal changes of TF expression over time. Trying to integrate data from different species when experimentally dissecting a GRN can, therefore, be potentially misleading, and so we focus here on data from *Xenopus*.

In *Xenopus*, most TFs expressed in the early ectoderm including many NPB specifiers are initially expressed very broadly throughout the ectoderm at blastula stages but become increasingly restricted to either the ventral (Dlx3/5, GATA2/3, Vent1/2, FoxI1/3, AP2 and Msx1), or the dorsal ectoderm (Zic1-5, Sox3) during gastrulation [10–20]. Zic TFs are subsequently downregulated in the central neural plate during gastrulation, while several other TFs such as Pax3 and Hairy2b are upregulated in a domain comprising the prospective NC and lateral neural plate [14, 21]. The ventral to dorsal BMP gradient, which is established during gastrulation due to the dorsal secretion of BMP antagonists from the axial mesoderm (organizer) plays a major role in establishing the ventrally or dorsally restricted expression of these TFs. Whereas many of the ventrally restricted TFs such as Dlx3/5, Msx1, GATA2/3, AP2, FoxI1/3, and Vent1/2 have been shown to be directly or indirectly activated by BMPs, most of the dorsally restricted TF including Sox3 and Zic genes are repressed by BMPs [11, 12, 22–26].

While dorsally and ventrally restricted TFs are broadly overlapping in the intermediate ectoderm at the beginning of gastrulation, the region of overlap decreases more and more and the boundaries between TF expression domains sharpen. At the end of gastrulation Dlx3/5, GATA2/3 and FoxI1/3 TFs are confined to the ventral, non-neural ectoderm (prospective epidermis and PPE), whereas Zic1, Pax3 (with the exception of a small domain in the prospective profundal placode) and Hairy2 are confined to a complementary region in the dorsal, neural ectoderm (prospective NC and neural plate) [14, 15, 21]. Vent1/2, AP2 and Msx1 are also ventrally restricted but their expression extends further dorsal than Dlx3/5 into the prospective NC forming domain, where they continue to overlap with Zic1 and Pax3 [16, 19, 27, 28].

Although the role of many early ectodermal TFs for NC specification has been well characterized, we know very little about their role in the specification of the PPE and in the segregation of PPE and NC territories during gastrulation. Previous studies have suggested that some of the ventrally restricted TFs in particular Dlx3/5, GATA2/3, AP2 and FoxI1/3 act as non-neural competence factors. These are required for the adoption of epidermal and PPR fates and promote the adoption of one or the other non-neural fate in a signaling dependent manner [15, 24, 29] with BMP inhibition and Wnt inhibition in combination with FGF signaling being required for PPE induction [30–32]. Recent studies in chick embryos have provided new insights into the temporal hierarchy of TF expression during PPE formation [33, 34] and have shown that TFs which later become confined to neural plate, neural crest and PPE are initially coexpressed in many cells at the NPB [35]. However, how these and other early ectodermal TFs affect PPE versus NC specification at the NPB is currently unknown.

In the present study, we use microinjections of mRNAs and Morpholino antisense oligonucleotides (MOs) into embryos of *Xenopus laevis* to systematically explore how seven early ectodermal TFs—the ventrally restricted FoxI1a, Vent2 (= Ventx2), Msx1, and AP2 (= TFAP2) and the dorsally restricted Zic1, Pax3 and Hairy2b (= Hes4)—affect the establishment of PPE (*Six1*, *Eya1*), NC (*FoxD3*) and neural plate territories (*Sox3*) during gastrulation. We use additional gain and loss of function studies of the PPE specifier genes Six1 and Eya1 to elucidate feedback regulation on these early ectodermal TFs. Our findings reveal a complex GRN resplendent with positive and negative feedback loops at the developing NPB and provide novel insights into how separate PPR and NC territories are established during gastrulation.

## Results

### All NPB TFs are required for PPE formation

**Fig. 1:**
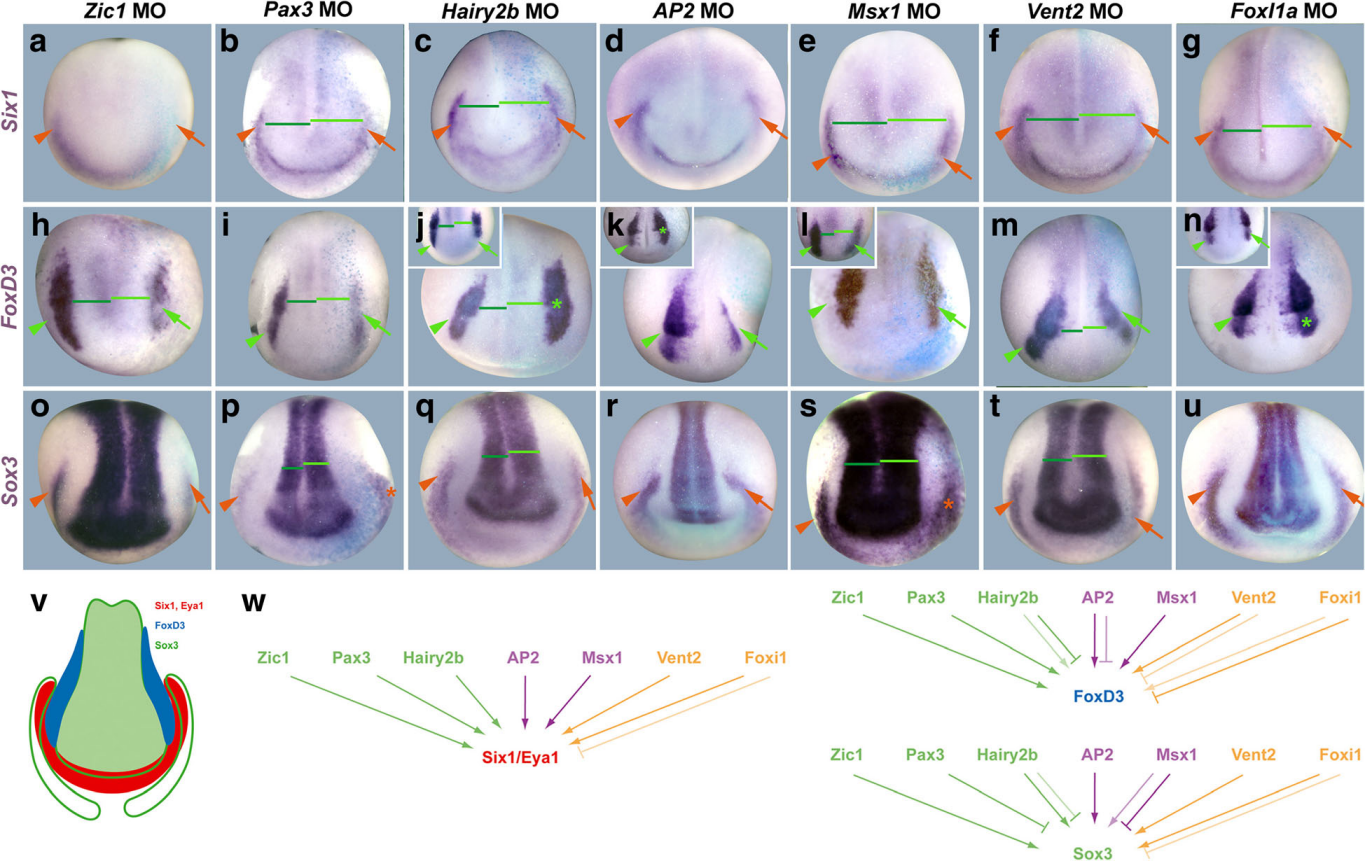
Requirement of early ectodermal TFs for PPE and NC formation. **a**–**u** Expression of PPE (*Six1*, *Sox3*), NC (*FoxD3*) and neural plate (*Sox3*) markers in dorsal views of neural plate stage *Xenopus* embryos after injection of MOs blocking translation of early ectodermal TF genes. Anterior is to the bottom. Control side is shown on the left and injected side on the right (as indicated by blue LacZ staining). Reductions (arrows) and increased or ectopic expression domains (asterisks) in the neural (green) and non-neural ectoderm (orange) compared with the control side (arrowheads) are indicated. Green lines highlight broadening of the neural plate and lateral displacement of NPB markers on the injected side (bright green) versus control side (dark green). Insets show alternative phenotypes. **v** Schematized gene expression domains of *Six1*/*Eya1* (PPE: red), *FoxD3* (NC: blue) and *Sox3* (neural plate and PPE: green outlines) in a neural plate stage *Xenopus* embryo (dorsal view, anterior to the bottom). *Sox3* expression in neural plate is indicated by green filling. Modified from [27]. **w** Summary of regulatory interactions**.** Arrows indicate requirement of TFs for expression of Six1/Eya1, FoxD3 or PPE Sox3 (reduction after TF knockdown). Bars indicate requirement of TF for restriction of expression of Six1/Eya1, FoxD3 or Sox3 (increase after TF knockdown). Faint colors indicate less frequent phenotypes. See Additional file 1: Table S3 for numbers

To elucidate which of the early ectodermal TFs are required for the proper establishment of PPE and NC territories, we first investigated how MO-mediated knockdown of these TFs affects *Six1*, *Eya1*, *Sox3* and *FoxD3* expression at the NPB. The efficacy and specificity of all MOs used has been validated in previous studies which included rescue experiments of various genes expressed at the neural plate border (Additional file 1: Table S1). Moreover, with the exception of Hairy2b and Vent2 (for which no orthologous genes are known in rodents), mutants in genes encoding these TFs (FoxI1, Msx1, AP2, Pax3, Zic1) in mouse and/or zebrafish are perturbed, like morphants, in NPB-derived tissues (Additional file 1: Table S1). Similar to previous studies, we initially injected high doses of MOs (10–20 ng) for each TF. This resulted in strong reduction of *Six1* and *Eya1* expression in the PPE of most embryos after knockdown of each TF (Additional file 1: Table S2). To reduce the probability of unspecific side effects of MOs, we then performed a more extensive analysis of MO knockdown phenotypes after injecting much lower doses (1–2 ng) of these MOs. Even at these low doses, MOs perturbed NPB development at a relatively high frequency; however, the phenotypes tended to be less severe than after injection of higher doses (e.g. resulting in relatively mild rather than strong reduction of NPB marker expression) (Fig. 1, Additional file 2: Figure S1, Additional file 1: Table S3).

*Six1* and *Eya1* expression in the PPE was downregulated in a high proportion of embryos after knockdown of both the dorsally restricted TFs Zic1, Pax3 and Hairy2b and the ventrally restricted TFs AP2, Vent2 and FoxI1a and in a smaller proportion after knockdown of the ventrally restricted Msx1 (Fig. 1a–g, Additional file 2: Figure S1, Additional file 1: Table S3). This indicates that all of these TFs are required for establishing *Six1* and *Eya1* expression in the PPE. *Sox3* expression in the PPE was also reduced after knockdown of most TFs but rarely or never after Pax3 and Msx1 knockdown (Fig. 1o–u, Additional file 1: Table S3). Whether this reduction of Sox3 is due to a direct requirement of these TFs for placodal *Sox3* expression or an indirect consequence of the downregulation of *Six1* and *Eya1* in the absence of these TFs remains to be determined. In contrast to other TFs, Msx1 and Pax3 loss of function typically resulted in the expansion of Sox3 expression from the PPE into adjacent non-neural ectoderm and this was occasionally also observed after Hairy2b and FoxI1a knockdown. Taken together this suggests a requirement of Zic1, AP2 and Vent2 (and to some extent Hairy2b and FoxI1a) in activating *Sox3* expression in the PPE, whereas Msx1 and Pax3 (and to some extent Hairy2b and FoxI1a) are mostly required for repression of *Sox3* throughout the non-neural ectoderm.

Knockdown of each TF also led to reduction of *FoxD3* expression in the NC in at least some embryos (Fig. 1h–n, Additional file 1: Table S3). However, only Zic1, Pax3, Msx1 and Vent2 loss of function showed reduction of *FoxD3* in the majority of embryos. In contrast, after Hairy2b, AP2 and FoxI1a loss of function, *FoxD3* was reduced only in some embryos but was increased in others. This suggests that while each TF is required for establishing *FoxD3* expression in the NC, Hairy2b, AP2 and FoxI1a play additional roles for restricting *FoxD3* expression to the NC domain in distinct and partly counteracting pathways.

Finally, *Sox3* expression in the neural plate was broadened (and the expression domains of *FoxD3*, *Six1* and *Eya1* were laterally displaced) in most embryos after knockdown of Pax3, Msx1 and Vent2 and in a minority of embryos (and usually only mildly) after knockdown of Zic1, Hairy2b and FoxI1a (Fig. 1o–u, Additional file 1: Table S3) suggesting that these TFs—in particular Pax3, Msx1 and Vent2—contribute to define the lateral limit of *Sox3* expression in the neural plate.

### The dorsally restricted TFs Zic1 and Pax3 are cell-autonomously required for PPE formation

In our knockdown experiments, MOs were injected at 2–8 cell stages and, thus, potentially could exert their effects by blocking translation of their target mRNAs in all germ layers. Since Msx1 and Vent2 are expressed in both mesoderm and ectoderm during gastrulation and early neural plate stages [11, 36], we can thus not rule out that some of the deficiencies in NPB marker expression after knockdown of Msx1 or Vent2 may reflect mesodermal rather than ectodermal functions of these genes in NPB formation. In contrast, Zic1, Pax3, Hairy2b, AP2 and FoxI1 are predominantly ectodermally expressed during gastrulation and early neural plate stages [27], suggesting that the phenotypes observed reflect a function of these TFs in the embryonic ectoderm.

However, during gastrulation, Zic1 and Pax3 become confined to a dorsal, neural ectodermal territory with a progressively decreasing degree of overlap and increasingly sharper boundary with the expression domains of Six1 and Eya1 in the PPE or with expression of Dlx3, GATA2 or FoxI1a in the non-neural ectoderm [14, 15, 19, 27]. This raises the possibility that Zic1 and Pax3 may be non-cell-autonomously required for PPE formation in the adjacent neural plate (e.g. by promoting the formation of signaling molecules required for PPE formation). To determine whether Zic1 and Pax3 are cell-autonomously required for PPE formation in the presumptive PPE ectoderm (presumably before the end of gastrulation when expression domains still overlap) or are instead required in the adjacent neural plate, we grafted the neural plate from embryos injected with Zic1 MO or Pax3 MO orthotopically into uninjected embryos or vice versa (Additional file 3: Figure S2), thereby juxtaposing Zic1 MO- or Pax3 MO-injected neural plate ectoderm with uninjected ectoderm in the PPE region. Control experiments with grafts from GFP-injected embryos showed that the grafting procedure itself did not affect *Six1* expression (Additional file 3: Figure S2 A). Similarly, no reduction of *Six1* expression was observed after grafting neural plates from Zic1 MO- or Pax3 MO-injected embryos into uninjected hosts (Additional file 3: Figure S2 B, D). Conversely, grafting neural plates from uninjected embryos into Zic1MO- or Pax3MO-injected embryos was unable to rescue reductions of *Six1* expression observed in the host PPE (Additional file 3: Figure S2 C, E). Taken together, this indicates that both Zic1 and Pax3 are required cell-autonomously for PPE formation.

### AP2 and Msx1 are sufficient to promote PPE markers in neural ectoderm

**Fig. 2:**
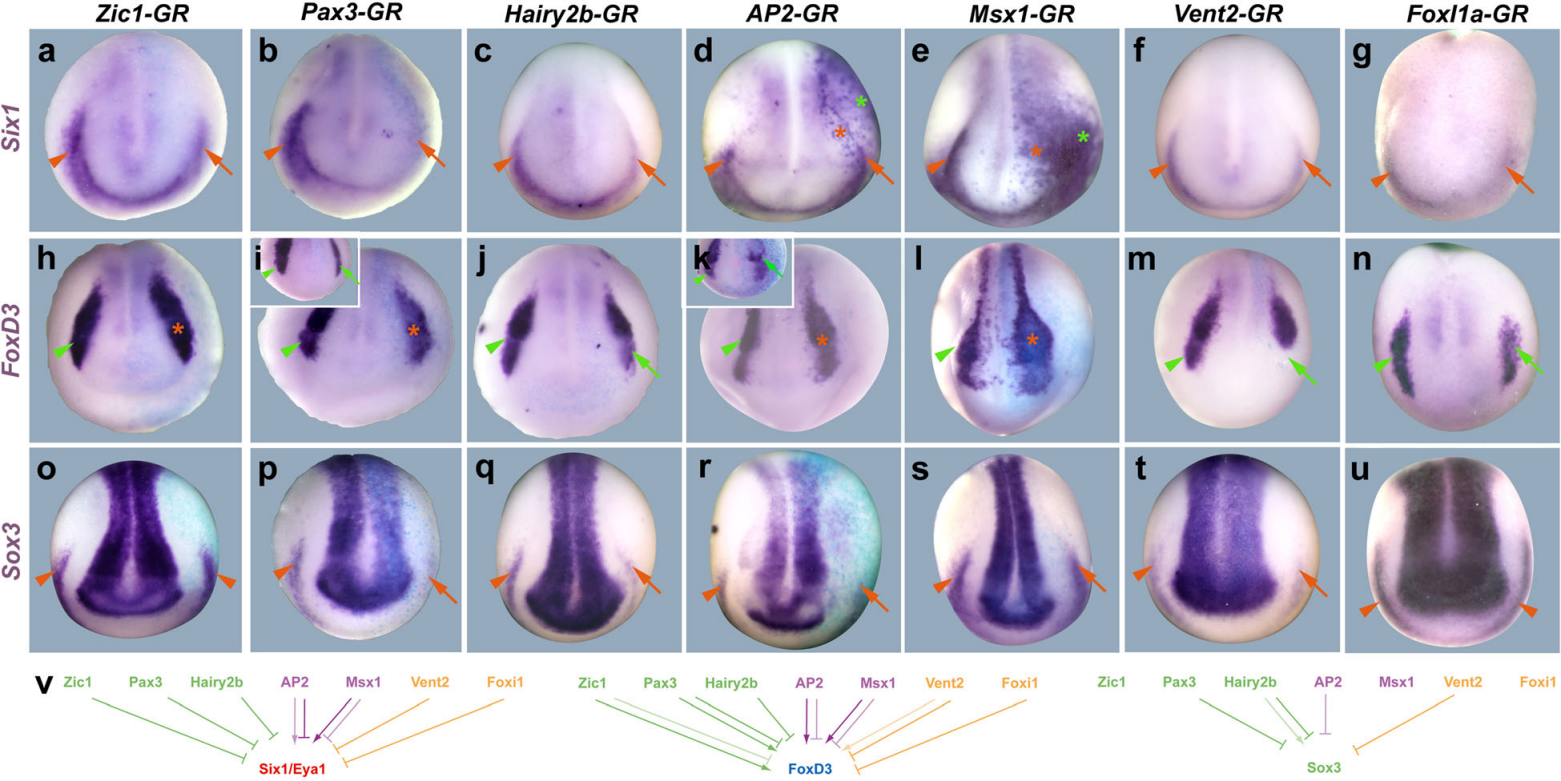
Role of early ectodermal TFs for PPE and NC formation. **a**–**u** Expression of PPE (*Six1*, *Sox3*), NC (*FoxD3*) and neural plate (*Sox3*) markers in dorsal views of neural plate stage *Xenopus* embryos after injection of mRNAs for hormone-inducible early ectodermal TF genes and dexamethasone activation from stage 11–12. Anterior is to the bottom. Control side is shown on the left and injected side on the right (as indicated by blue LacZ staining). Reductions (arrows) and increased or ectopic expression domains (asterisks) in the neural (green) and non-neural ectoderm (orange) compared with the control side (arrowheads) are indicated. Insets show alternative phenotypes. **v** Summary of regulatory interactions. Arrows indicate ability of TFs to promote expression of Six1/Eya1, FoxD3 or Sox3. Bars indicate ability of TF to repress Six1/Eya1, FoxD3 or PPE Sox3. Faint colors indicate less frequent phenotypes. See Additional file 1: Tables S4 and S5 for numbers

We next tested whether overexpression of any of the early ectodermal TFs is sufficient to promote the activation of PPE or NC markers. Since injection of mRNAs encoding these TFs (Additional file 1: Table S4) often affected early development and may lead to gastrulation defects (especially for Hairy2b and Vent2), we also injected hormone-inducible constructs of TFs, which were activated by dexamethasone treatment at the end of gastrulation (Fig. 2a–g, Additional file 4: Figure S3, Additional file 1: Table S5). Overexpression of all dorsally restricted TFs, Zic1, Pax3 and Hairy2b, reduced *Six1* and *Eya1* expression in the PPE. Pax3, in particular, resulted in very strong and often complete repression of *Six1* or *Eya1*, while Zic1 and Hairy2b had milder effects. Overexpression of the ventrally restricted TFs also led to occasional reductions of *Six1* and *Eya1* expression (most frequently for AP2 and Vent2). However, overexpression of AP2 and Msx1 also promoted ectopic expression of *Six1* and *Eya1* not only in the non-neural ectoderm but also in the neural plate similar to what was previously described after Dlx3 overexpression [15]. This suggests that AP2 and Msx1 play a central role in PPE formation possibly by endowing ectoderm with non-neural ectodermal competence as previously shown for AP2 in zebrafish [29]. *Sox3* expression in the PPE was reduced after the overexpression of Pax3, Hairy2b, AP2 and Vent2 but unaffected by Zic1, Msx1 or Vent2 overexpression (Fig. 2o–u, Additional file 1: Table S5) indicating that its regulation in the PPE depends on different combinations of TF than *Six1* or *Eya1*.

The effects of overexpression of most TFs on the NC were more complex and variable. While overexpression of each TF led to reduced *FoxD3* expression in a subset of embryos, overexpression of each TF except AP2 and Msx1 also led to the expansion of *FoxD3* expression (but never to ectopic expression) in another subset of embryos (Fig. 2h–n, Additional file 1: Table S5). Taken together, this suggests that these TFs act in a complex and combinatorial fashion to promote NC expression.

*Sox3* expression in the neural plate was reduced in scattered cells after overexpression of Pax3, Msx1 and Vent2 (Fig. 2o–u, Additional file 1: Table S5) in accordance with the proposed role of these TFs in defining the lateral border of neural *Sox3* expression.

### AP2, Msx1 and Dlx3 promote PPE formation via different pathways

**Fig. 3:**
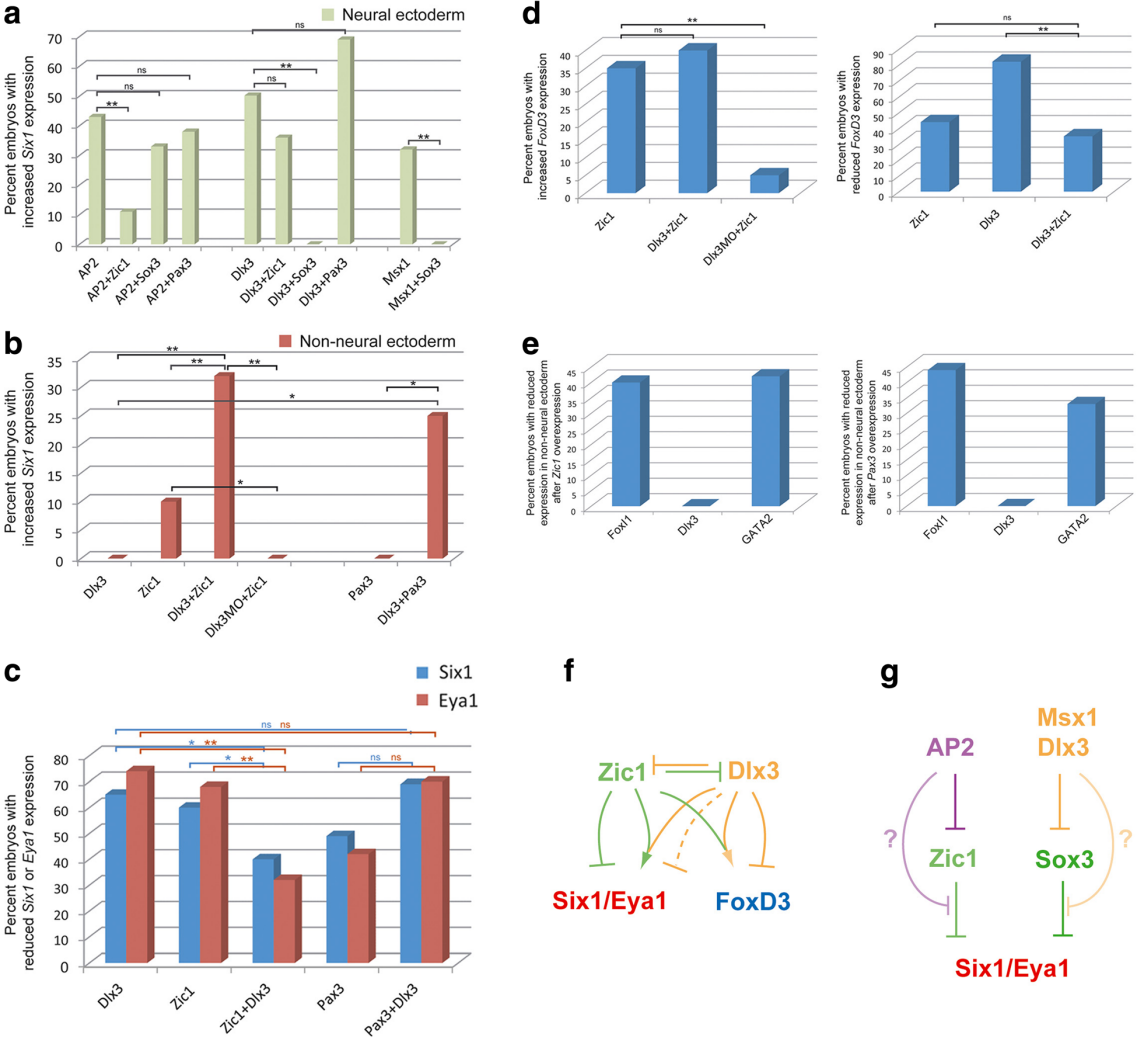
Cooperation of early ectodermal TFs in PPE and NC formation. **a**–**d** Cooperative effects of TFs as revealed in coinjection experiments. Significant differences are indicated (two-tailed Fisher’s exact test; *p < 0.05, **p < 0.001; ns: not significant). See Additional file 1: Tables S4 and S6 for numbers. **e** Reduction of TF expression in non-neural ectoderm after overexpression of Zic1 or Pax3. See Additional file 1: Table S7 for numbers. **f**, **g** Summary of regulatory interactions**.** Arrows indicate positive transcriptional regulation. Bars indicate negative transcriptional regulation. Solid lines are based on both loss and gain of function data, while hatched lines are supported only by gain of function data. Faint lines with question marks in **g** indicate potential alternative pathways for AP2 and Msx1/Dlx3

The observation that many dorsally restricted TFs including Zic1 and Pax3 (see above) but also Sox3 (Additional file 1: Table S6) repress *Six1* and *Eya1* expression in the PPE suggests that the ability of AP2, Msx1 and Dlx3 [15] to ectopically activate *Six1* and *Eya1* in the neural plate may depend on their ability to repress some or all dorsally restricted TFs (Msx1 and Dlx3 repress Sox3: see above and [15]; AP2 represses Zic1: see [19]). To test this, we determined whether coinjection of Zic1, Pax3 or Sox3 could prevent ectopic neural expression of *Six1* after AP2, Dlx3 or Msx1 injection (Fig. 3a, b; Additional file 1: Table S6). The frequency of ectopic neural *Six1* expression was indeed significantly reduced after coinjection of AP2 with Zic1 (but not with Sox3 or Pax3) or coinjection of Dlx3 or Msx1 with Sox3 (but not with Zic1 or Pax3 in the case of Dlx3; these were not tested for Msx1) (Fig. 3a; Additional file 1: Table S6). This suggests that AP2 and Dlx3/Msx1 promote PPE formation in neural ectoderm via different pathways, viz. by inhibition of Zic1 and Sox3, respectively. Indeed, Sox3 immunostaining in vibratome sections of embryos in which *Six1* was ectopically expressed in the neural plate after overexpression of Dlx3 or Msx1, shows that Sox3 is specifically reduced in the injected part of the neural plate in which *Six1* is ectopically expressed (Additional file 5: Figure S4). Whether Zic1 is similarly reduced in the area of AP2 overexpression remains to be determined once a specific antibody recognizing *Xenopus* Zic1 becomes available.

### Zic1 and Pax3 promote PPE formation only in Dlx3-expressing ectoderm

While coinjection of Zic1 or Pax3 with Dlx3 does not significantly alter the frequency of ectopic *Six1* expression in the neural ectoderm, it significantly increases the frequency of ectopic *Six1* expression in the non-neural ectoderm compared to injection of either Dlx3 or Pax3 alone, which never promote non-neural *Six1* expression or to Zic1 alone, which promotes *Six1* only in a small subset of embryos (Fig. 3b, Additional file 1: Table S6). Conversely, coinjection of Dlx3 MO with Zic1 completely blocks the ability of Zic1 to promote *Six1* expression (Fig. 3b Additional file 1: Table S6). This suggests that Zic1 and Pax3 can promote *Six1* only in Dlx3-expressing ectoderm. Coinjection of Zic1 (but not Pax3) and Dlx3 also significantly reduces the frequency of decreased *Six1* or *Eya1* expression in the PPE compared to overexpression of Zic1 or Dlx3 alone (Fig. 3c, Additional file 1: Table S6) suggesting that the combination of both TFs protects against the repressive effect of each TF alone.

Similarly, coinjection of Dlx3 MO with Zic1 significantly reduces the ability of Zic1 to promote *FoxD3* expression (Fig. 3d, Additional file 1: Table S6). However, Dlx3 overexpression represses *FoxD3* at high frequency, which is significantly reduced by coinjection of Zic1 (Fig. 3d, Additional file 1: Table S6). Taken together, this indicates that Zic1 also requires Dlx3 for NC formation, and protects *FoxD3* from repression by Dlx3.

Our results demonstrate that Zic1 and Pax3 are required for the cell-autonomous activation of *Six1* in the PPE but do so only in conjunction with Dlx3. However, Dlx3 and another ventrally restricted TF GATA2 were previously shown to repress Zic1 and Pax3 [15]. Taken together, this suggests that dorsally restricted TFs Zic1 and Pax3 may be required for the initiation of PPE formation in Dlx3-expressing ectoderm but subsequently become excluded from the Dlx3-expressing part of the ectoderm. To determine whether cross-repressive interactions contribute to the sharpening of the boundary between non-neural ectoderm expressing the ventrally restricted TFs Dlx3, GATA2 and FoxI1a and neural ectoderm expressing Zic1 and Pax3, we injected Zic1 and Pax3 and analysed the effect on Dlx3, GATA2 and FoxI1a expression (Fig. 3e, Additional file 1: Table S7). While FoxI1a and GATA2 expression was reduced, Dlx3 was not affected indicating that Zic1 and Pax3 indeed repress some but not all ventrally restricted TFs.

### Cross-regulation of NPB TFs by Six1 and Eya1

**Fig. 4:**
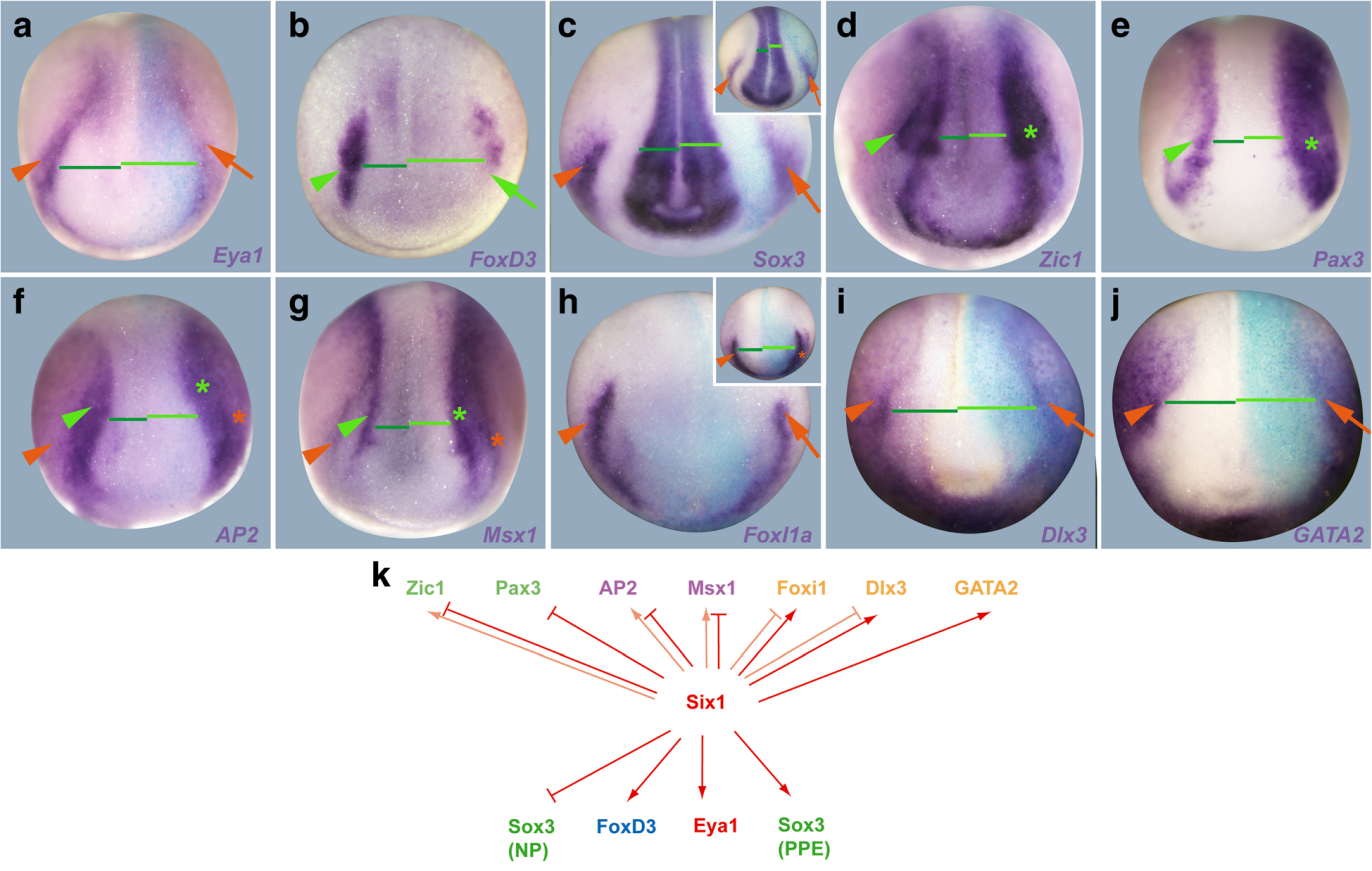
Effects of Six1 knockdown on early ectodermal TFs. **a**–**j** Expression of PPE (*Eya1*, *Sox3*), NC (*FoxD3*), neural plate (*Sox3*) markers and early ectodermal TFs in dorsal views of neural plate stage *Xenopus* embryos after injection of Six1 MO1 + MO2. Anterior is to the bottom. Control side is shown on the left and injected side on the right (as indicated by blue LacZ staining). Reductions (arrows) and increased or ectopic expression domains (asterisks) in the neural (green) and non-neural ectoderm (orange) compared with the control side (arrowheads) are indicated. Green lines highlight broadening of the neural plate and lateral displacement of NPB markers on the injected side (bright green) versus control side (dark green). Insets show alternative phenotypes. **k** Summary of regulatory interactions. Arrows indicate requirement of Six1 for expression of TFs (reduction after Six1 knockdown). Bars indicate requirement of Six1 for restriction of expression of TFs (increase after Six1 knockdown). Faint colors indicate less frequent phenotypes. See Additional file 1: Table S8 for numbers

**Fig. 5:**
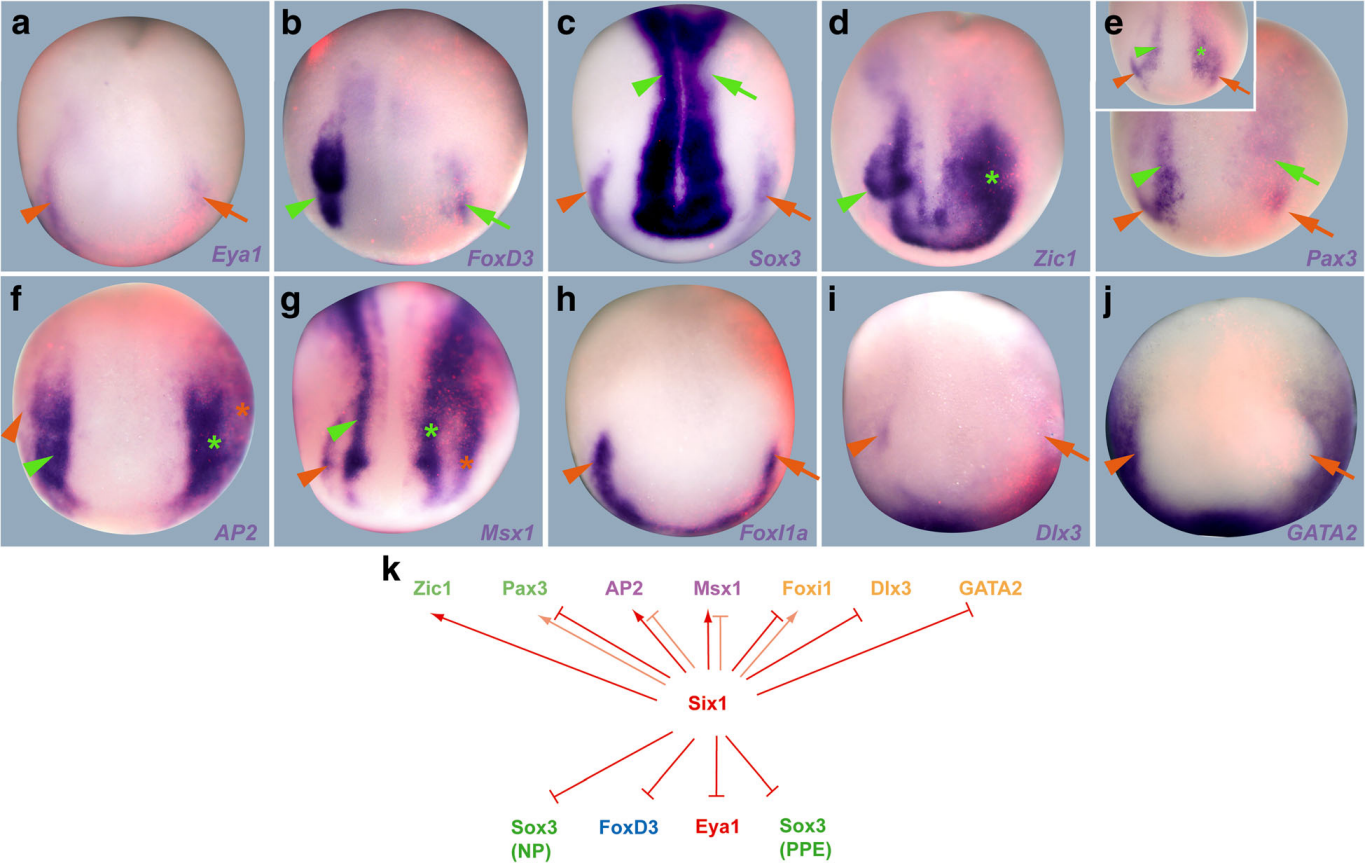
Effects of Six1 overexpression on NPB markers and other ectodermal TFs. **a**–**j** Expression of PPE (*Eya1*, *Sox3*), NC (*FoxD3*), neural plate (*Sox3*) markers and early ectodermal TFs in dorsal views of neural plate stage *Xenopus* embryos after injection of Six1 mRNA. Anterior is to the bottom. Control side is shown on the left and injected side on the right (as indicated by red LacZ immunostaining). Arrowheads indicate expression domains on the control side. Reductions (arrows) and increased or ectopic expression domains (asterisks) in the neural (green) and non-neural ectoderm (orange) compared with the control side (arrowheads) are indicated. Insets show alternative phenotypes. **k** Summary of regulatory interactions**.** Arrows indicate ability of Six1 to promote expression of TFs. Bars indicate ability of Six1 to repress TFs. Faint colors indicate less frequent phenotypes. See Additional file 1: Table S9 for numbers

We finally analysed the expression of NPB TF genes (*Zic1*, *Pax3*, *AP2*, *Msx1*, *FoxI1a*, *Dlx3* and *GATA2*) as well as dedicated PPE (*Six1*, *Eya1*), NC (*FoxD3*) and neural plate markers (*Sox3*) using injection of Six1 and Eya1 MOs (Fig. 4, Additional file 6: Figure S5, Additional file 1: Table S8) and mRNAs (Fig. 5, Additional file 7: Figure S6, Additional file 1: Table S9) to determine whether Six1 and Eya1 cross-regulate these other TFs. Again, the efficacy and specificity of the Six1 and Eya1 MOs used has been validated in previous studies (Additional file 1: Table S1). Since Six1 and Eya1 MOs were injected at 2–8 cell stages, we cannot completely rule out that some of the observed phenotypes reflect early embryonic or non-ectodermal roles of Six1 and Eya1. However, up to neural plate stages expression of both genes is largely confined to the NPB ectoderm as well as to a domain in the paraxial mesoderm, which is much more medial and posterior than the NPB [37, 38] suggesting that the deficits observed in the NPB after Six1 or Eya1 knockdown reflect mostly their ectodermal function.

Knockdown of either Six1 or Eya1 leads to reductions of *Eya1*, *Six1* and *Sox3* expression in the PPE; reductions of *FoxD3* in the NC; lateral displacement of *Six1*, *Eya1* and *FoxD3*; and broadening of *Sox3* expression in the neural plate (Fig. 4a–c, Additional file 6: Figure S5 A-C, Additional file 1: Table S8). This suggests that Six1 and Eya1 themselves are required for PPE as well as NC formation. It remains possible that gastrulation defects (impaired convergence-extension), which are sometimes observed after knockdown of Six1 or Eya1 contribute to the observed shift of the neural plate border. However, lateral displacement of PPE domains of *Eya1* or *Six1* after Six1 or Eya1 knockdown, respectively, was also observed in embryos with relatively normal *Six1* or *Eya1* expression in the paraxial mesoderm (which should also be affected by gastrulation defects), suggesting that Six1 and Eya1 also play a more direct role in setting the lateral border of the neural plate.

To gain insights into how Six1 and Eya1 modulate the establishment of different ectodermal territories at the NPB, we also analysed the effects of Six1 and Eya1 knockdown on earlier ectodermal TFs. Knockdown of either Six1 or Eya1 slightly reduces the level of expression for genes encoding ventrally restricted TFs *FoxI1a*, *Dlx3* and *GATA2* and shifts their expression boundaries laterally (Fig. 4, Additional file 6: Figure S5, Additional file 1: Table S8) suggesting that Eya1 and Six1 appear to be required for the maintenance of high-level expression of ventrally restricted TFs in the PPE. Conversely, knockdown of either Six1 or Eya1 results in broader and stronger expression of *Zic1*, *Pax3*, *AP2* and *Msx1* in the neural plate and NC (Fig. 4, Additional file 6: Figure S5, Additional file 1: Table S8). This indicates that Six1 and Eya1 are required for repressing and laterally delimiting *Zic1*, *Pax3*, *AP2* and *Msx1* at the NPB, thereby helping to confine strong expression of these TFs to the NC.

We next analysed the effect of Six1 or Eya1 overexpression at the NPB. Overexpression of Eya1 often broadens *Six1* and *Sox3* expression in the non-neural ectoderm (although it reduces non-neural Sox3 expression in another subset of embryos) and promotes *Six1* even ectopically in the neural plate (Additional file 7: Figure S6, Additional file 1: Table S9). It also results in increased or ectopic *FoxD3* expression in NC and neural plate, but causes reduction of *Sox3* expression in the neural plate suggesting that Eya1 promotes both PPE and NC but represses dedicated neural plate markers. While Six1 overexpression causes similar but less pronounced reductions of *Sox3* in the neural plate than Eya1, it leads to reductions of *Eya1* in the PPE and of *FoxD3* in the NC, different from Eya1. Taken together, this suggests that Six1 despite being required for PPE and NC formation similar to Eya1 negatively regulates NPB markers in additional, Eya1-independent pathways. The ability of Six1 to interact not only with the coactivator Eya1 but also alternatively with corepressors [30] may at least partly account for these effects although this has to be confirmed in further studies.

Overexpression of Eya1 and Six1 causes a reduction of expression of some genes encoding ventrally restricted TFs such as *Dlx3* and *GATA2* expression, whereas, overexpression of Eya1 causes an increase in *FoxI1a* expression and overexpression of Six1 has variable effects on *FoxI1a* (Fig. 5, Additional file 7: Figure S6, Additional file 1: Table S9). Thus, while our knockdown experiments indicated that Eya1 and Six1 appear to be required for the maintenance of ventrally restricted TFs, high levels of Six1 and Eya1 seem to repress *Dlx3* and *GATA2*.

Somewhat paradoxically, overexpression of Eya1 and Six1 has rather similar effects on NC-enriched TFs *Zic1*, *Pax3*, *AP2* and *Msx1* than Six1 or Eya1 knockdown generally resulting in broadening and stronger expression in the neural plate and NC with the exception that Six1 (but not Eya1) overexpression typically resulted in repression of *Pax3*, whereas Eya1 (but not Six1) overexpression led to reduced *Msx1* expression (Fig. 5, Additional file 7: Figure S6). Thus, while our knockdown experiments demonstrate that Six1 and Eya1 are both required (possibly in a cooperative fashion) for repressing and laterally delimiting Zic1, Pax3, AP2 and Msx1 in the NC, these overexpression experiments indicate that they act as inhibitors of these TFs only in certain contexts, for example only in cooperation with other cofactors or in a dosage dependent way. Moreover, while Six1 and Eya1 may jointly promote *Zic1* and *AP2*, they independently promote *Msx1* and *Pax3*, respectively, presumably in conjunction with other binding partners.

## Discussion

### NPB TFs are required for PPE and NC formation in a context-dependent fashion

Previous studies have implicated both dorsally (e.g. Zic1, Pax3, Hairy2b) and ventrally restricted (e.g. Dlx3, AP2, Msx1) “NPB specifiers” together with BMP, Wnt and FGF signals in NC specification [14, 18, 19, 21, 39–42]. Moreover, ventrally restricted TFs Dlx3, GATA2, FoxI1a and AP2 were shown to be essential for endowing ectoderm with the competence to form PPE in response to BMP and Wnt inhibitors and FGF signals [15, 24, 29]. Here we confirm and extend these observations in demonstrating that all early ectodermal TFs analysed are required for both PPE and NC formation. However, expression of Zic1 and Pax3 can be inferred to be non-overlapping with Six1 expression at neural plate stages [10, 27, 39] and Zic1 was recently shown to be able to promote *Six1* expression at a distance [43]. This raises the possibility that these dorsally restricted TFs may promote PPE formation non-cell autonomously by being required in the neural plate for the production of signals contributing to induction of the PPE in adjacent non-neural ectoderm. However, we show here that both Zic1 and Pax3 are required cell-autonomously for PPE formation but that they promote PPE formation only in the presence of Dlx3, a ventrally restricted non-neural competence factor. In the absence of Dlx3, Zic1 instead antagonizes PPE formation, thereby preventing expression of PPE markers. While high levels of Dlx3 alone are also capable of repressing *Six1* and *Eya1*, this may be prevented in the developing PPE by the negative feedback regulation of *Dlx3* expression by Six1 and Eya1 themselves. With decreasing overlap between ventral, Dlx3 expressing and dorsal, Zic1 expressing territories during gastrulation, PPE markers, thus, become confined to the non-neural, Dlx3-expressing ectoderm during gastrulation.

Furthermore, we show here that Zic1 only promotes *FoxD3* in the presence of Dlx3 and that Zic1 neutralizes the repressive effect of Dlx3 on *FoxD3* expression. This suggests that similarly to PPE formation, NC markers are initially upregulated in the region of overlap between Zic1 and Dlx3 but with increasing separation of Dlx3 expressing and Zic1 expressing territories during gastrulation ultimately become restricted to a complementary region of ectoderm, viz. the neural ectoderm, devoid of Dlx3 expression.

### Ventrally restricted TFs promote PPE formation via separate pathways

Previous studies have shown that the ventrally restricted TFs Dlx3, AP2, GATA2/3 and FoxI1a act as non-neural competence factors which can promote PPE marker expression when overexpressed in the neural plate [15, 24, 29]. We here confirm this for AP2 in *Xenopus* and demonstrate that Msx1 is also able to promote neural Six1 and Eya1 expression, while FoxI1a is unable to do so different from zebrafish. Our coinjection experiments further suggest that ventrally restricted TFs promote *Six1* and *Eya1* expression in the neural ectoderm by counteracting dorsally restricted TFs and that different non-neural TFs act by counteracting different neural TFs with AP2 antagonizing Zic1 and Dlx3 and Msx1 antagonizing Sox3. Indeed, AP2 was previously shown to repress Zic1 expression [19], and we find in the present study that Dlx3 and Msx1 repress Sox3, confirming previous studies showing repressive effects of Dlx3, Dlx5 and Msx1 on Sox2 or Sox3 in the neural plate [15, 41, 42, 44, 45]. Taken together with the context-dependent activity of Zic1 discussed above, these findings are compatible with a scenario in which coexpression of both Zic1 and Sox3 is required to block PPE formation so that the repressive effect of Dlx3 on Sox3 expression may at least partly account for the ability of Dlx3 to counteract the inhibitory action of Zic1 on PPE formation. However, additional experiments are required to establish conclusively, whether the effects of AP2 and Dlx3/Msx1are due mainly to the direct or indirect repression of Zic1 and Sox3 by AP2 and Dlx3/Msx1, respectively [15, 19], or whether AP2 and Dlx3/Msx1 also act downstream of Zic1 and Sox3, respectively, by neutralizing their inhibitory effects on PPE formation. The fact that different non-neural TFs act via different pathways may underlie the synergistic effects between different non-neural competence factors previously reported [24], although it remains to be confirmed whether AP2 and Dlx3/Msx1 also act synergistically.

### Dynamic interactions of TFs in the NPB region lead to separation of PPE and NC territories

Two different models have previously been proposed to explain the formation of immediately juxtaposed PPE and NC territories at the NPB (Additional file 8: Figure S7 A). The neural plate border state model suggests that initially a NPB region is formed between prospective neural plate and epidermis, from which subsequently PPE is induced laterally and NC medially [3–5, 7]. The binary competence model instead proposed that the ectoderm first becomes subdivided into a ventral, non-neural competence territory and a dorsal, neural competence territory and that the PPE can only be induced from the former, whereas NC can only be induced from the latter [15, 31]. Our present findings suggest a new perspective that is able to unite both models (Additional file 8: Figure S7 B).

In support of the binary competence model, data presented in this and a previous study [15] suggest that the PPE is ultimately confined to ventral (non-neural) ectoderm, expressing Dlx3 and other non-neural competence factors, whereas the NC is confined to dorsal (neural) ectoderm expressing Zic1. The separation between these two territories appears to be driven by cross-repressive interactions between dorsally restricted TFs Zic1 and Pax3 and ventrally restricted TFs Dlx3, GATA2 and FoxI1a. While Dlx3 and GATA2 have been previously shown to repress Zic1 and Pax3 [15], we here demonstrate that Zic1 and Pax3 in turn repress GATA2 and FoxI1a. It is worth noting that other ventrally restricted TFS AP2 and Msx1 do not respect the same boundary but extend further dorsal into Zic1/Pax3 expressing terrain, where they play essential roles in initiating the expression of NC specifier genes [18, 19, 41, 42]. We propose that the presence of Pax3 in this territory prevents upregulation of PPE markers despite the ability of AP2 to repress Zic1. As our findings here taken together with previous studies [14, 18, 41, 46] indicate, Zic1, Pax3 as well as Msx1 and Vent2 also repress Sox3 in the neural ectoderm while Vent2 is in turn repressed by Sox3 [16]. These TFs may, thus, cooperate to define the boundary between NC and neural plate territories.

In support of the neural plate border state model, we present evidence that both dorsally and ventrally restricted TFs are required for both PPE and NC formation presumably during early gastrulation when these two classes of TFs still overlap broadly. During gastrulation, this region of overlap, the NPB region, becomes smaller and smaller probably in response to cross-repressive interactions between TFs and possibly shifting BMP concentrations. Hence the dynamically shrinking NPB region is the region of the embryo which retains the overlapping expression of dorsally and ventrally restricted TFs that is seen throughout the entire ectoderm at the beginning of gastrulation for the longest period of time. It is tempting to speculate that the combination of these TFs may keep the NPB region in an early embryonic regulatory state and facilitate the maintenance of a network of pluripotency factors precisely in this region until the end of gastrulation, as recently demonstrated [47].

It is important to note, however, that from this perspective, the NPB is not an individualized area with a unique regulatory state, where common progenitors of NC and PPE are defined by expression of “multilineage selector” TFs that would then be shared between the different lineages arising from these progenitors, similar to the retina, where Pax6 is required for development of all retinal cell lineages [48]. Rather, the NPB is an area of indecision or of “multilineage priming” [49], where common progenitors of NC and PPE are defined by the co-expression of lineage determining TFs, which will later segregate to different lineages, similar to what has been shown for the hematopoietic system [50]. This interpretation is supported by a recent study in the chick demonstrating that individual NPB cells co-express TFs associated with neural plate (Sox2), NC (Pax7) and PPE (Six1) in various combinations before these TF domains become segregated to different cell populations around neural tube closure [35]. Importantly, we show here that some of the TFs that become ultimately excluded from a particular ectodermal lineage (e.g. Zic1 from the PPE and Dlx3 from the NC) are nevertheless required for its differentiation, suggesting that the transient co-expression of TFs in its early progenitors is not merely permissive but plays an important regulatory role.

Does the transient co-expression of different lineage specific TFs in single cells at the NPB reflect a tree-like regulatory hierarchy of binary cell fate decisions between cell lineages or rather a more network-like situation where a particular cell lineage can arise in alternative pathways from different progenitors with different combinations of TFs? In the absence of solid information about which TFs act as lineage determinants, no definitive answer can be given. However, in the first case, NPB cells co-expressing TFs of neural plate (NP), neural crest (NC), PPE and epidermis would be expected to give rise to more restricted progenitors only co-expressing TFs’ characteristic for NP-NC or PPE-E (as predicted by the binary competence model) or for NC-PPE (as predicted by the neural plate border state model) before committing to one of the ectodermal cell lineages, whereas in the second case alternative progenitors co-expressing other combinations of TFs (e.g. NP-NC-PPE or NP-PPE) should exist. The recent study of Roellig and coauthors provides evidence for the heterogeneity of NPB cells revealing the presence of cells co-expressing all different combinations of TFs (Sox2-Pax7-Six1; Sox2-Pax7; Pax7-Six1, Sox2-Six1; epidermal TFs were not analysed) [35]. However, this study also shows that the majority of cells (44–58%) co-express Sox2 and Pax7 (but not Six1), while only 1–4% coexpress Six1 and Pax7 (but not Sox2) and only 0–1% coexpress Sox2 and Six1 (but not Pax7). This predominance of NP-NC over NC-PPE progenitors supports a regulatory hierarchy as predicted by the binary competence model (even though epidermal TFs were not analysed precluding the identification of common PPE-E progenitors). Whether the rare occurrence of progenitors exhibiting Six1-Pax7 or Sox2-Six1 co-expression indicates the existence of alternative pathways to regulate lineage restrictions at the NPB or reflect stochastic fluctuations of TF levels or differences in protein degradation rates should be resolved in further studies combining single-cell sequencing with functional experiments establishing the role of various TFs in lineage specification.

### Formation of PPE and NC territories involves complex feedback regulation by Six1 and Eya1

Our findings provide evidence that Six1 and Eya1 are not only required for stabilizing their own expression in the PPE but are also required for FoxD3 expression in the NC. Although in line with a previous study [30] we observe lateral expansion of the *FoxD3* expressing domain after Six1 MO injections, levels of *FoxD3* expression are substantially reduced in the majority of embryos. This indicates an unexpected role of Six1 and Eya1 in NC formation, which is also supported by the observed increase of *FoxD3* after Eya1 overexpression. Eya1 and Six1 are also required for maintenance of ventrally restricted TFs Dlx3, GATA2 and FoxI1a and for delimiting lateral expression of Zic1, Pax3, AP2 and Msx1 expression but the regulatory relationships are complex and await further clarification.

The phenotypes of embryos after Six1 and Eya1 loss or gain of function are usually similar in accordance with the well-documented synergistic action of both proteins as TF (Six1) and coactivator (Eya1) [51, 52]. However, we observed some notable differences in the regulation of *FoxD3* (upregulated by Eya1, repressed by Six1), *Pax3* (upregulated by Eya1, repressed by Six1) and *Msx1* (upregulated by Six1, repressed by Eya1). This suggests that Six1 and Eya1 affect these genes at least partly via different pathways. In support of this, a previous study has shown that Six1 promotes reduction of *FoxD3* together with groucho corepressors [30], but further studies are needed to elucidate alternative pathways for Eya1 and the pathways involved in the regulation of Pax3 and Msx1 by Six1.

### A new model for NPB development

**Fig. 6:**
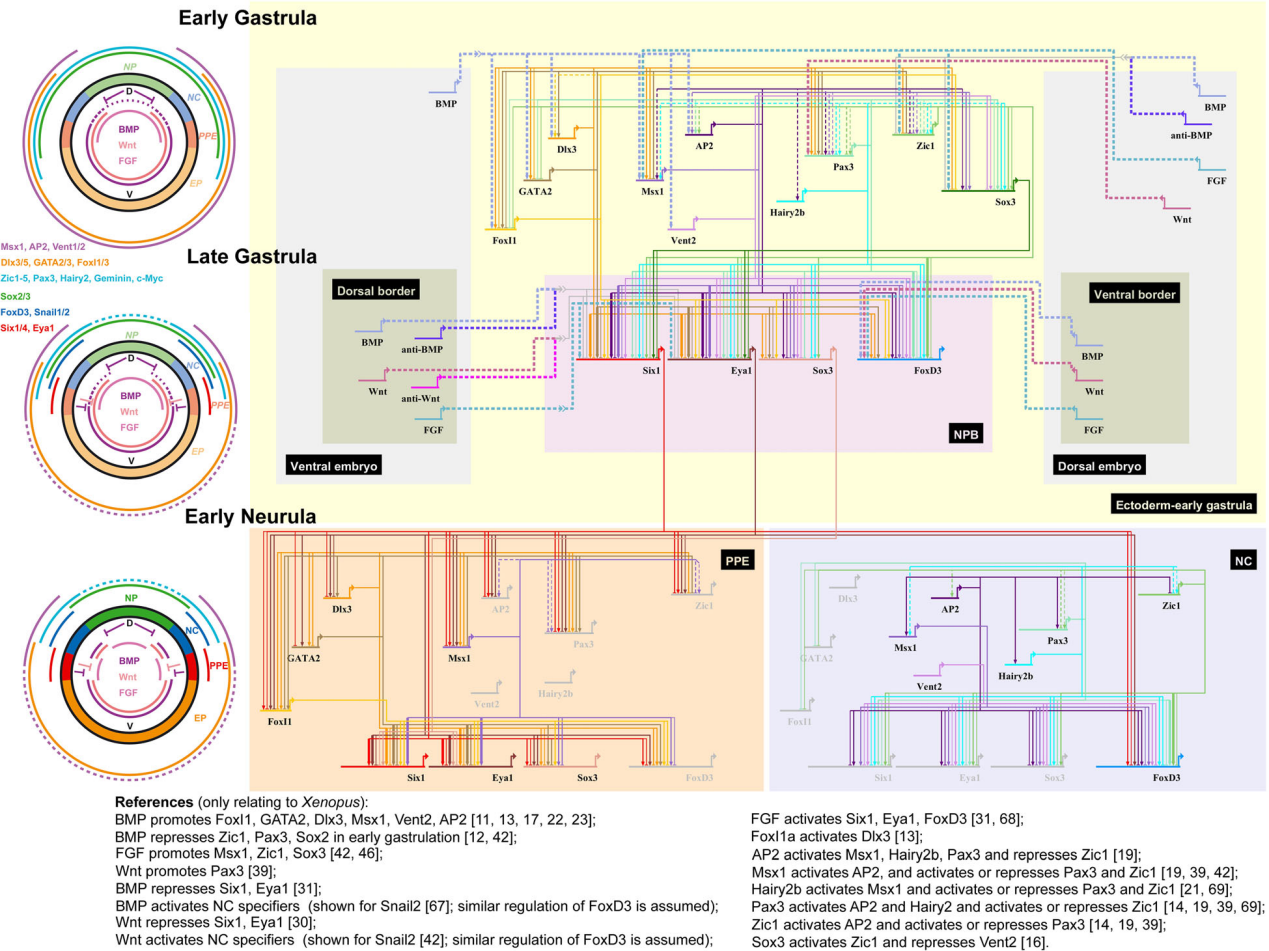
Gene regulatory network (GRN) for NPB development in *Xenopus laevis*. Greyed out genes represent those inactive in a particular cell population (e.g. Zic1 in the PPE). All interactions shown are based on functional studies and may be direct or indirect. Solid lines indicate relationships established in the present study or in [15], whereas hatched lines indicate relationships established in previous studies referenced below. Arrows indicate activation. Bars show repression. Thick lines indicate relationships verified in loss of function (and often also in gain of function) experiments, while thin lines indicate relationships only supported by gain of function experiments. Signaling pathways are shown in extra thick lines. Often there is experimental evidence to support both activation and repression of genes by upstream TFs. Further studies are needed to elucidate the interactions determining these context-dependent effects. In the absence of functional data, temporal changes of regulatory relationships are proposed here based on changing expression patterns. Panels on the left depict idealized cross sections through cranial region of *Xenopus* embryos showing TF distribution at three stages of development (D, dorsal; V, ventral) (modified from [2]). Hatched lines indicate downregulation of expression. Pax3 and c-Myc TFs only get upregulated in the lateral part of the turquoise domain during gastrulation. Presumptive neural plate (NP), neural crest (NC), preplacodal ectoderm (PPE), and epidermis (EP) are shown as fate map for gastrula stages (faint colors) and as specified territories for the early neurula (strong colors). BMP, Wnt, and FGF signaling is shown by colored lines inside the schematized embryo, with graded BMP activity and approximate position of sources of BMP inhibitors and Wnt inhibitors indicated (bars). During gastrulation, many TFs become increasingly dorsally (turquoise and green) or ventrally (orange and pink) restricted, and the region of overlap decreases. NC (blue) and PPE (red) specifiers become confined to non-overlapping territories in the neural (turquoise) and non-neural (orange) ectoderm at the end of gastrulation. See text for details

**Fig. 7:**
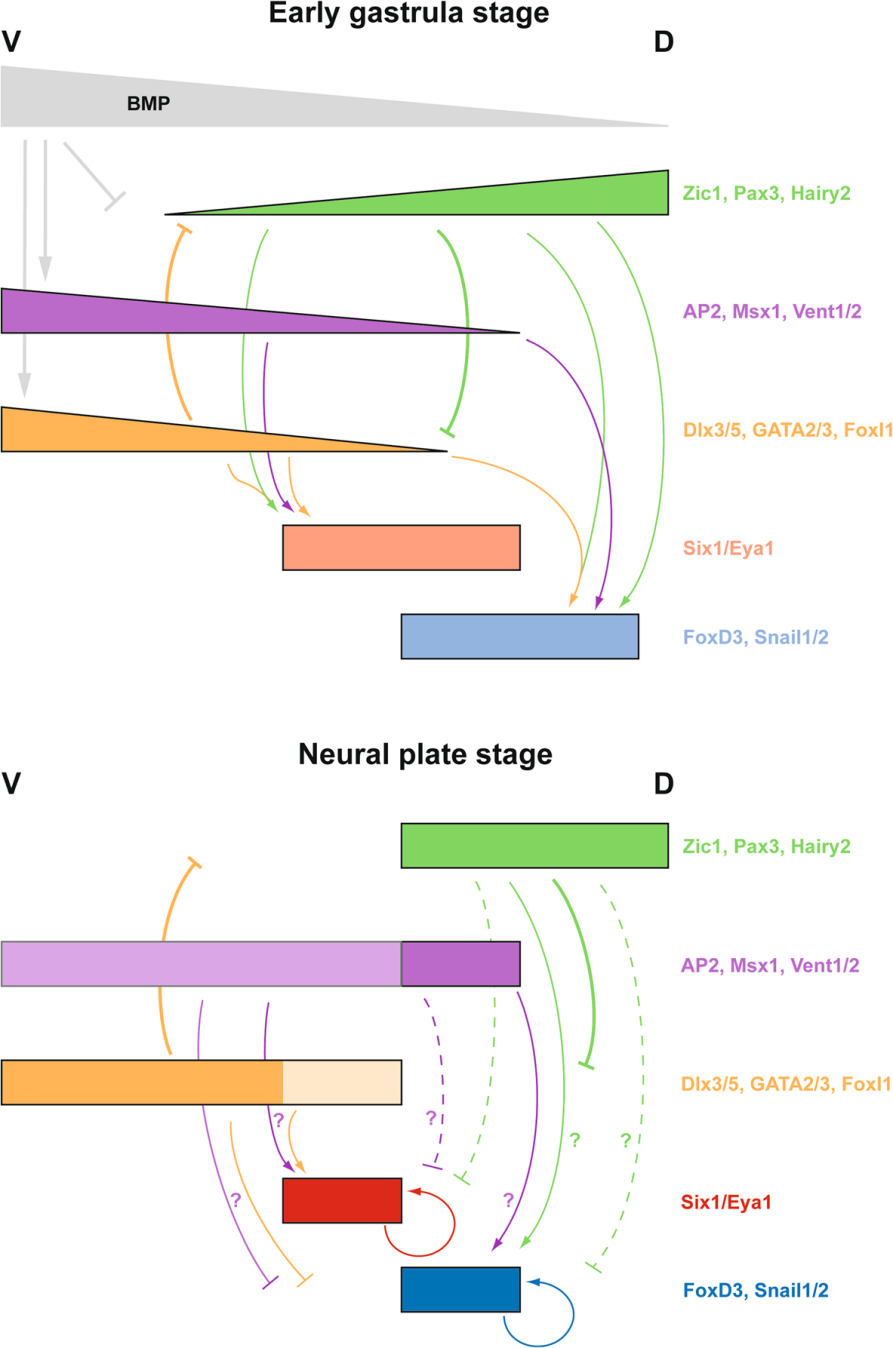
A model for the establishment of PPE and NC at the NPB. Spatial distribution of expression levels of TFs in the ectoderm from dorsal (D) to ventral (V) is shown at the beginning of gastrulation (upper panel) and after completion of gastrulation (lower panel). Faint colors indicate weak expression. TFs are grouped according to shared expression patterns. BMP activity levels are also shown for the early stage. Arrows indicate positive regulatory relationships, while lines with bars indicate negative regulatory relationships. Solid lines indicate relationships that have been verified in loss of function (and usually also in gain of function) experiments, while hatched lines indicate relationships that are only supported by gain of function experiments. Temporal changes of regulatory relationships are proposed based on changing TF expression patterns.The context-dependent regulation of Six1 and Eya1 as well as of FoxD3 by Dlx3 and Zic1 is shown by converging arrows. The question marks in the lower panel indicate that TFs probably cooperate with other as yet unknown factors in a context-dependent way to either activate or repress target genes. Cross-regulation of TFs by Six1 and Eya1 is not depicted for clarity. See text for details

Our comprehensive functional analysis of the role of TFs in establishing different ectodermal territories at the NPB together with findings from previous studies allows us to draft a detailed GRN addressing the formation of PPE and NC territories at the NPB (Fig. 6) and to propose a new model for NPB development (Fig. 7). Due to the currently very limited information about the binding of TFs to enhancers of target genes, we do not know in most cases whether the depicted regulatory relationships are direct or indirect. Furthermore, in the absence of solid experimental evidence about temporal changes in regulatory relationships over time, we propose temporal changes of regulatory relationships based on changing TF expression patterns.

In response to the developing ventrodorsal gradient of BMP and additional signaling events at the beginning of gastrulation, expression domains of many ectodermal TFs become either dorsally (e.g. Zic1, Sox3, Pax3, Hairy2b) or ventrally restricted (Dlx3, GATA2, AP2, Msx1, Vent2). Many dorsally restricted TFs are inhibited by BMP and thus progressively downregulated ventrally, while many ventrally restricted TFs are activated by BMP and thus progressively downregulated dorsally as a consequence of dorsal BMP inhibition by signals from the organizer. Among the ventrally restricted TFs, expression of AP2, Msx1 and Vent2 extends further dorsally than expression of Dlx3, GATA2 and FoxI1a.

Positive regulatory feedback loops between Dlx3, GATA2 and FoxI1a, between AP2 and Msx1 and between Pax3 and Zic1 stabilize the expression of these TF, whereas cross-repressive interactions between the dorsally restricted TFs Zic1 and Pax3 and ventrally restricted TFs Dlx3, GATA2 and FoxI1a (possibly aided by dynamic changes in the BMP gradient) result in a progressively decreasing degree of overlap between dorsally and ventrally restricted TFs during gastrulation. In the region of overlap (NPB), all TFs are required to initiate expression of PPE (*Six1*, *Eya1*) and NC specifiers (e.g. *FoxD3*) in an initially overlapping pattern. However, at least some dorsally restricted TFs such as Zic1 and ventrally restricted TF such as Dlx3 need to act cooperatively to promote PPE and NC formation. In the absence of Zic1, Dlx3 instead represses expression of NC specifiers (*FoxD3*), whereas in the absence of Dlx3, Zic1 represses PPE specifiers (*Six1*). Together with the cross-repression between Zic1 and Dlx3, this results in the ultimate confinement of the PPE to the Dlx3 expressing (non-neural) side and of the NC to the Zic1 expressing (neural) side of the ectoderm. Additional ventrally restricted TFs such as AP2, Msx1 and Vent2, which do not respect the boundary between neural and non-neural ectoderm, are also required for the upregulation of both NC specifiers and PPE specifiers, but additionally act to delimit their respective territories. The cooperative interaction partners underlying these context-dependent effects still remain to be identified.

Finally, positive auto- and cross-regulation between Six1 and Eya1 stabilize the PPE [30, 52, 53], while positive cross-regulation between different NC specifiers stabilizes the NC region [7]. Six1 and Eya1 also contribute to the definition of boundaries between expression domains of various TFs by both positive and negative feedback regulation.

## Conclusion

In summary, our present study in Xenopus embryos reveals a detailed gene regulatory network at the neural plate border upstream of preplacodal ectoderm formation based on functional interactions between ectodermal transcription factors. Additional studies focusing on cis-regulatory regions will be required to determine which of these interactions are direct. We propose a new model to explain the formation of immediately juxtaposed preplacodal ectoderm and neural crest territories at the neural plate border, uniting previous models

## Methods

### Expression constructs

Expression constructs used in the present study are listed in Additional file 1: Table S10 together with injection dosage used and references. Plasmids encoding hormone-inducible constructs of FoxI1a and Zic1 were newly constructed here. To generate pCS2^+^-GR-FoxI1a, we PCR amplified the GR insert from pCS2-GR-Pax3 with forward and reverse primers containing KpnI and SacI restriction sites, respectively (forward: 5′-GGTACCGCAGGATCCCATCGATTCGA-3′; reverse: 5′-GAGCTCTGGATCTACGTAATACGACTCACT-3′) and after restriction digestion subcloned the PCR fragment upstream of FoxI1a into pCS2^+^-xFoxi1a. To generate pCS2^+^-GR-Zic1, we PCR amplified the GR insert from pCS2-GR-Pax3 with forward and reverse primers containing BamHI and ClaI restriction sites, respectively (forward: 5′-GGATCCGCAGGATCCCATCGATTCGA-3′; reverse: 5′-ATCGATTGGATCTACGTAATACGACTCACT-3′) and after restriction digestion subcloned the PCR fragment upstream of Zic1 into pCS2^+^-Zic1. The sequences were confirmed by sequencing.

### Morpholinos

Translation blocking morpholino antisense oligonucleotides (MO) against *Six1*, *Eya1*, *Zic1*, *Pax3*, *Hairy2a*, *Hairy2b*, *AP2*, *Msx1*, *FoxI1a*, *Vent2* and *Dlx3* were previously described and their efficacy and specificity were verified as indicated in Additional file 1: Table S1. Sequences and references are given in Additional file 1: Table S1. A standard control MO (5′-CCTCTTACCTCAGTTACAATTTATA-3′) obtained from GeneTools was used in control injections.

### Microinjections

Embryos of *Xenopus laevis* were obtained by hormone-induced egg laying followed by in vitro fertilization or natural matings, staged according to [54] and injected according to standard procedures [55]. Capped mRNAs were synthesized with Message Machine Kit (Ambion) and injected into single blastomeres at the 2- to 4-cell stage that give rise to the dorsal ectoderm. The dosage of injected mRNAs is given in Additional file 1: Table S10. MOs against *Zic1*, *Pax3*, *AP2*, *Msx1*, *FoxI1a* and *Vent2* (see above) were injected singly and MOs against *Hairy2a* and *Hairy2b* as a cocktail into single blastomeres at the 2–8-cell stage (1–2 ng for each MO). In accordance with previous studies [15, 30, 56], higher amounts of Dlx3 MO and cocktails of Six1 MO1 + Six1 MO2 or Eya1 MO1 + Eya1 MO2 were injected (10–20 ng for each MO). Co-injection of *myc-GFP* (125 pg; pCMTEGFP kindly provided by Doris Wedlich) or *lacZ* (250 pg) identified the injected side. For activation of hormone-inducible constructs, embryos were incubated in dexamethasone (10 μM; Sigma) from stages 11–13 onwards.

### In situ hybridization and immunohistochemistry

Embryos injected with *myc-GFP* were sorted under a fluorescent stereomicroscope and were then fixed according to standard procedures [55]. *LacZ*-injected embryos were fixed and then stained with X-Gal to reveal lacZ. Wholemount in situ hybridization was carried out under high stringency conditions at 60 °C as previously described [27] using digoxigenin-labelled antisense probes against *Eya1* [37], *Six1* [38], *FoxD3* [57], *Sox3* [58], *Zic1* [10], *Pax3* [59], *AP2* [18], *Msx1* [60], *FoxI1a* [61], *GATA2* [62], *Dlx3* [63]. Each marker was analysed using embryos from at least three different batches of eggs from different females. After in situ hybridization, myc-tagged proteins were revealed immunohistochemically using mouse anti-c-myc antibody (9E10, Developmental Studies Hybridoma Bank) as previously described [31]. In some embryos, lacZ distribution was revealed immunohistochemically using a polyclonal rabbit anti-LacZ (MP Biomedicals Cappel, Santa Ana, California; Cat.: 55976; 1:1000) and an Alexa594-conjugated anti-rabbit antibody (1:1000).

Vibratome sections (30–40 μm) were prepared after whole mount in situ hybridization [27]. Sox3 was revealed immunohistochemically in sections using anti-Sox3 (1:1000) primary antibodies [64] and anti-rabbit-Alexa594 conjugated secondary antibodies (Invitrogen; 1:500 each), as previously described [27]. Nonspecific binding of secondary antibodies was not observed when primary antibodies were omitted in control reactions.

### Data analysis

Embryos were included in the analysis, whenever unilateral lacz staining was apparent (even when lacz staining was weak or confined to the ventral side). To determine changes in marker expression after microinjection of mRNAs or MOs, injected sides of embryos were always scored relative to the control side of the same embryo. To determine whether marker expression was reduced or increased, in each embryo, the total level of marker gene expression was compared by eye between injected and control side. To ensure consistency of scoring, all embryos were scored by the same researcher (not blinded). Since embryos sometimes displayed complex phenotypes with reduced level of marker expression in one part of the neural or non-neural ectoderm but increased level of marker expression in other parts, reductions and increases were assessed separately in each embryo and were treated as independent and not mutually exclusive categories. In all images, control sides of embryos are shown on the left and injected sides on the right. Reductions (arrows) and increased or ectopic expression domains (asterisks) in the neural (green) and non-neural ectoderm (orange) compared with the control side (arrowheads) are indicated.

In knockdown experiments, it was also quantified separately, whether the expression domains of marker genes were laterally or medially displaced on the injected side compared to the control side. To determine displacement, in each embryo, the distance from the dorsal midline to the dorsal border of marker gene expression domain on the injected side was compared by eye to the distance from the dorsal midline to the marker gene expression on the control side. While displacements of expression domains were also observed in gain of function experiments, this was not quantified, since displacements were generally much milder after injection of hormone-inducible constructs suggesting that they may mostly reflect unspecific effects on gastrulation movements. In images, the width of the neural plate and the distance of NPB markers from the dorsal midline are highlighted by green lines (bright green on the injected side versus dark green on the control side).

Some embryos could only be checked for reduced expression, for increased expression, or for displacements, and numbers analysed for the different categories of phenotypes are, thus, not always identical. BioTapestry [65, 66] was used to depict regulatory interactions as a GRN (Fig. 6).

### Grafting experiments

Embryos were injected either with myc-GFP mRNA (125 pg) alone (control) or in combination with Pax3 MO or Zic1 MO (10–20 ng each) into both blastomeres at the 2-cell stage. At stage 13, the left lateral part of the anterior neural plate (corresponding to region LNP of [31]) was then grafted orthotopically from injected donor embryos to uninjected host embryos and vice versa. Grafting procedures were performed as previously described [31]. Embryos were then fixed in 4% paraformaldehyde at stage 15 for expression analysis by in situ hybridization.

## Additional files

Additional file 1: **Table S1.** Sequences and validation of Morpholinos used. **Table S2.** Changes in Six1 and Eya1 expression in the non-neural (placodal, epidermal) ectoderm after injection of various MO at high levels. **Table S3.** Changes in marker gene expression in the non-neural (placodal, epidermal), and neural ectoderm after the injection of various MO. **Table S4.** Changes in marker gene expression in the non-neural (placodal, epidermal), and neural ectoderm after the injection of various mRNAs for early TFs. **Table S5.** Changes in marker gene expression in the non-neural (placodal, epidermal), and neural ectoderm after the injection of various mRNAs for hormone-inducible early TFs. **Table S6.** Changes in marker gene expression in the non-neural (placodal, epidermal), and neural ectoderm after the co-injection of various mRNAs. **Table S7.** Changes in marker gene expression in the non-neural (placodal, epidermal), and neural ectoderm after the injection of *Zic1* and *Pax3* mRNAs. **Table S8.** Changes in marker gene expression in the non-neural (placodal, epidermal), and neural ectoderm after knockdown of Six1 or Eya1. **Table S9.** Changes in marker gene expression in the non-neural (placodal, epidermal), and neural ectoderm after overexpression of Six1 or Eya1. **Table S10.** Doses of mRNAs injected. (DOCX 131 kb)

Additional file 2: **Figure S1.** Requirement of early ectodermal TFs for *Eya1* expression in the PPE. Expression of PPE marker *Eya1* in dorsal views of neural plate stage *Xenopus* embryos after injection of MOs blocking translation of early ectodermal TF genes. Anterior is to the bottom. Control side is shown on the left and injected side on the right (as indicated by blue LacZ staining). Reductions in the non-neural ectoderm (orange arrows) compared with the control side (orange arrowheads) are indicated. Green lines indicate broadening of the neural plate and lateral displacement of NPB markers on the injected side (bright green) versus control side (dark green). See Additional file 1: Table S3 for numbers. (PDF 254 kb)

Additional file 3: **Figure S2.** Cell autonomous requirements of Zic1 and Pax3 for PPE formation. Neural plates were orthotopically grafted from donor embryos to host embryos. A: Control grafts from GFP injected embryos into uninjected hosts. There is no effect on *Six1* expression in the PPE (except for a slight decrease in 1/4 embryos). B: Grafting a neural plate from Pax3 MO injected embryo into uninjected hosts does not affect *Six1* expression in the PPE (except for 1/10 cases). C: A neural plate graft from an uninjected embryo is unable to rescue deficits in *Six1* expression in the PPE (arrow) of Pax3 MO injected embryos evident in 2/4 embryos. D: Grafting a neural plate from Zic1 MO injected embryo into uninjected hosts does not affect *Six1* expression in the PPE (0/5). E: A neural plate graft from an uninjected embryo is unable to rescue deficits in Six1 expression in the PPE (arrow) of Zic1 MO injected embryos evident in 2/5 embryos. Asterisk indicates *Six1* expression in graft. Arrowheads indicate the *Six1* expression domain in the PPE on the control side. G: graft. (PDF 2727 kb)

Additional file 4: **Figure S3.** Role of early ectodermal TFs for *Eya1* expression in the PPE. Expression of PPE marker *Eya1* in dorsal views of neural plate stage *Xenopus* embryos after injection of mRNAs for hormone-inducible early ectodermal TF genes and dexamethasone activation from stage 11–12. Anterior is to the bottom. Control side is shown on the left and injected side on the right (as indicated by blue LacZ staining). Reductions (arrows) and increased or ectopic expression domains (asterisks) in the neural (green) and non-neural ectoderm (orange) compared with the control side (arrowheads) are indicated. See Additional file 1: Tables S4 and S5 for numbers. (PDF 261 kb)

Additional file 5: **Figure S4.** *Six1* and Sox3 expression after overexpression of *Dlx3* or *Msx1*. Transverse sections through neural plate or neural tube of *Xenopus* embryos after injection of *Dlx3* (A) or *Msx1* (B) mRNA and in situ hybridization for *Six1*. Sections are shown in brightfield (A_1_, B_1_) and in an overlay of red and UV fluorescent channels (A_2_, B_2_). LacZ (turquoise in A1 and B1) reveals the extent of mRNA injection in the neural plate (hatched outlines). Nuclei are stained by DAPI (blue). Sox3 immunopositive nuclei are shown in pink. Ectopic *Six1* expression is confined to *Dlx3*- or *Msx1*-injected regions of the neural plate, which lack Sox3 immunoreactivity. Abbreviations: not, notochord, np: neural plate, nt: neural tube, PPE: preplacodal ectoderm, so: somite. Bar: 50 μm (for all panels). (PDF 659 kb)

Additional file 6: **Figure S5.** Effects of Eya1 knockdown on NPB markers and other ectodermal TFs. A-J: Expression of PPE (*Six1*, *Sox3*), NC (*FoxD3*), neural plate (*Sox3*) markers and early ectodermal TFs in dorsal views of neural plate stage *Xenopus* embryos after injection of Eya1 MO1 + MO2. Anterior is to the bottom. Control side is shown on the left and injected side on the right (as indicated by blue LacZ staining). Arrowheads indicate expression domains on the control side. Reductions (arrows) and increased or ectopic expression domains (asterisks) in the neural (green) and non-neural ectoderm (orange) compared with the control side (arrowheads) are indicated. Green lines indicate broadening of the neural plate and lateral displacement of NPB markers on the injected side (bright green) versus control side (dark green). Insets show alternative phenotypes. K: Summary of regulatory interactions**.** Arrows indicate requirement of Eya1 for expression of TFs (reduction after Eya1 knockdown). Bars indicate requirement of Eya1 for restriction of expression of TFs (increase after Eya1 knockdown). Faint colors indicate less frequent phenotypes. See Additional file 1: Table S8 for numbers. (PDF 2013 kb)

Additional file 7: **Figure S6.** Effects of Eya1 overexpression on NPB markers and other ectodermal TFs. A-J: Expression of PPE (*Eya1*, *Sox3*), NC (*FoxD3*), neural plate (*Sox3*) markers and early ectodermal TFs in dorsal views of neural plate stage *Xenopus* embryos after injection of Eya1 mRNA. Anterior is to the bottom. Control side is shown on the left and injected side on the right (as indicated by blue LacZ staining). Reductions (arrows) and increased or ectopic expression domains (asterisks) in the neural (green) and non-neural ectoderm (orange) compared with the control side (arrowheads) are indicated. Green lines indicate broadening of the neural plate and lateral displacement of NPB markers on the injected side (bright green) versus control side (dark green). Insets show alternative phenotypes. K: Summary of regulatory interactions. Arrows indicate ability of Six1 to promote expression of TFs. Bars indicate ability of Six1 to repress TFs. Faint colors indicate less frequent phenotypes. See Additional file 1: Table S9 for numbers. (PDF 1906 kb)

Additional file 8: **Figure S7.** Models of placode specification. A: The “neural plate border state model” proposes that PPE (red) and NC (blue) are induced from a common precursor (purple) at the neural plate border, whereas the “binary competence model” proposes that they are induced from non-neural (yellow) and neural (green) ectodermal competence territories, respectively. B: In a new model that combines aspects of both these models, we propose here that there is indeed an NPB region during gastrulation, which gives rise to both PPE and NC. However, the NPB domain is not defined by a unique regulatory state but rather by the overlap of dorsally restricted neural (green) and ventrally restricted non-neural (yellow) competence factors (left panel; region of overlap: olive green). The degree of overlap decreases during gastrulation resolving into mutually exclusive non-neural and neural competence territories at the end of gastrulation (middle panel). Inducing signals from adjacent tissues induce preplacodal ectoderm (FGF, BMP-inhibitors, Wnt-inhibitors; red) and neural crest (FGF, BMP, Wnt; blue) at the border of non-neural and neural ectoderm, respectively (from [2]; modified from [70]). (PDF 2779 kb)

## References

[1] Northcutt RG, Gans C (1983). The genesis of neural crest and epidermal placodes: a reinterpretation of vertebrate origins. Q Rev Biol.

[2] Schlosser G. Early embryonic specification of vertebrate cranial placodes. WIREs Developmental Biology. 2014. 10.1002/wdev.142. 10.1002/wdev.142 25124756

[3] Saint-Jeannet JP, Moody SA (2014). Establishing the pre-placodal region and breaking it into placodes with distinct identities. Dev Biol.

[4] Grocott T, Tambalo M, Streit A (2012). The peripheral sensory nervous system in the vertebrate head: a gene regulatory perspective. Dev Biol.

[5] Meulemans D, Bronner-Fraser M (2004). Gene-regulatory interactions in neural crest evolution and development. Dev Cell.

[6] Milet C, Monsoro-Burq AH (2012). Neural crest induction at the neural plate border in vertebrates. Dev Biol.

[7] Simoes-Costa M, Bronner ME (2015). Establishing neural crest identity: a gene regulatory recipe. Development.

[8] Sauka-Spengler T, Bronner-Fraser M (2008). Evolution of the neural crest viewed from a gene regulatory perspective. Genesis.

[9] Schlosser G (2010). Making senses: development of vertebrate cranial placodes. IntRevCell MolBiol.

[10] Kuo JS, Patel M, Gamse J, Merzdorf C, Liu XD, Apekin V, Sive H (1998). opl: a zinc finger protein that regulates neural determination and patterning in *Xenopus*. Development.

[11] Suzuki A, Ueno N, Hemmati-Brivanlou A (1997). *Xenopus* msx1 mediates epidermal induction and neural inhibition by BMP4. Development.

[12] Mizuseki K, Kishi M, Matsui M, Nakanishi S, Sasai Y (1998). *Xenopus* zic-related-1 and sox-2, two factors induced by chordin, have distinct activities in the initiation of neural induction. Development.

[13] Matsuo-Takasaki M, Matsumura M, Sasai Y (2005). An essential role of *Xenopus* Foxi1a for ventral specification of the cephalic ectoderm during gastrulation. Development.

[14] Hong CS, Saint-Jeannet JP (2007). The activity of Pax3 and Zic1 regulates three distinct cell fates at the neural plate border. Mol Biol Cell.

[15] Pieper M, Ahrens K, Rink E, Peter A, Schlosser G (2012). Differential distribution of competence for panplacodal and neural crest induction to non-neural and neural ectoderm. Development.

[16] Rogers CD, Harafuji N, Cunningham DD, Archer T, Casey ES (2009). *Xenopus* Sox3 activates sox2 and geminin and indirectly represses Xvent2 expression to induce neural progenitor formation at the expense of non-neural ectodermal derivatives. MechDev.

[17] Luo T, Matsuo-Takasaki M, Thomas ML, Weeks DL, Sargent TD (2002). Transcription factor AP-2 is an essential and direct regulator of epidermal development in *Xenopus*. Dev Biol.

[18] Luo T, Lee YH, Saint-Jeannet JP, Sargent TD (2003). Induction of neural crest in *Xenopus* by transcription factor AP2alpha. Proc Natl Acad Sci U S A.

[19] de Croze N, Maczkowiak F, Monsoro-Burq AH (2011). Reiterative AP2a activity controls sequential steps in the neural crest gene regulatory network. Proc Natl Acad Sci U S A.

[20] Plouhinec JL, Medina-Ruiz S, Borday C, Bernard E, Vert JP, Eisen MB, Harland RM, Monsoro-Burq AH (2017). A molecular atlas of the developing ectoderm defines neural, neural crest, placode, and nonneural progenitor identity in vertebrates. PLoS Biol.

[21] Nichane M, de Croze N, Ren X, Souopgui J, Monsoro-Burq AH, Bellefroid EJ (2008). Hairy2-Id3 interactions play an essential role in *Xenopus* neural crest progenitor specification. Dev Biol.

[22] Feledy JA, Beanan MJ, Sandoval JJ, Goodrich JS, Lim JH, Matsuo-Takasaki M, Sato SM, Sargent TD (1999). Inhibitory patterning of the anterior neural plate in *Xenopus* by homeodomain factors Dlx3 and Msx1. Dev Biol.

[23] Friedle H, Knöchel W (2002). Cooperative interaction of Xvent-2 and GATA-2 in the activation of the ventral homeobox gene Xvent-1B. J Biol Chem.

[24] Kwon HJ, Bhat N, Sweet EM, Cornell RA, Riley BB (2010). Identification of early requirements for preplacodal ectoderm and sensory organ development. PLoS Genet.

[25] Marchal L, Luxardi G, Thome V, Kodjabachian L (2009). BMP inhibition initiates neural induction via FGF signaling and Zic genes. Proc Natl Acad Sci U S A.

[26] Rogers CD, Ferzli GS, Casey ES (2011). The response of early neural genes to FGF signaling or inhibition of BMP indicate the absence of a conserved neural induction module. BMC Dev Biol.

[27] Schlosser G, Ahrens K (2004). Molecular anatomy of placode development in *Xenopus laevis*. Dev Biol.

[28] Ladher R, Mohun TJ, Smith JC, Snape AM (1996). Xom: a *Xenopus* homeobox gene that mediates the early effects of BMP-4. Development.

[29] Bhat N, Kwon HJ, Riley BB (2012). A gene network that coordinates preplacodal competence and neural crest specification in zebrafish. Dev Biol.

[30] Brugmann SA, Pandur PD, Kenyon KL, Pignoni F, Moody SA (2004). Six1 promotes a placodal fate within the lateral neurogenic ectoderm by functioning as both a transcriptional activator and repressor. Development.

[31] Ahrens K, Schlosser G (2005). Tissues and signals involved in the induction of placodal Six1 expression in *Xenopus laevis*. Dev Biol.

[32] Litsiou A, Hanson S, Streit A (2005). A balance of FGF, BMP and WNT signalling positions the future placode territory in the head. Development.

[33] Trevers KE, Prajapati RS, Hintze M, Stower MJ, Strobl AC, Tambalo M, Ranganathan R, Moncaut N, Khan MAF, Stern CD (2018). Neural induction by the node and placode induction by head mesoderm share an initial state resembling neural plate border and ES cells. Proc Natl Acad Sci U S A.

[34] Hintze M, Prajapati RS, Tambalo M, Christophorou NAD, Anwar M, Grocott T, Streit A (2017). Cell interactions, signals and transcriptional hierarchy governing placode progenitor induction. Development.

[35] Roellig D, Tan-Cabugao J, Esaian S, Bronner ME. Dynamic transcriptional signature and cell fate analysis reveals plasticity of individual neural plate border cells. Elife. 2017;6:e21620. 10.7554/eLife.21620. 10.7554/eLife.21620 PMC5371430 28355135

[36] Onichtchouk D, Gawantka V, Dosch R, Delius H, Hirschfeld K, Blumenstock C, Niehrs C (1996). The xvent-2 homeobox gene is part of the bmp-4 signalling pathway controlling dorsoventral patterning of Xenopus mesoderm. Development.

[37] David R, Ahrens K, Wedlich D, Schlosser G (2001). *Xenopus* Eya1 demarcates all neurogenic placodes as well as migrating hypaxial muscle precursors. Mech Dev.

[38] Pandur PD, Moody SA (2000). Xenopus Six1 gene is expressed in neurogenic cranial placodes and maintained in differentiating lateral lines. Mech Dev.

[39] Sato T, Sasai N, Sasai Y (2005). Neural crest determination by co-activation of Pax3 and Zic1 genes in *Xenopus* ectoderm. Development.

[40] Milet C, Maczkowiak F, Roche DD, Monsoro-Burq AH (2013). Pax3 and Zic1 drive induction and differentiation of multipotent, migratory, and functional neural crest in *Xenopus* embryos. Proc Natl Acad Sci U S A.

[41] Tribulo C, Aybar MJ, Nguyen VH, Mullins MC, Mayor R (2003). Regulation of Msx genes by a bmp gradient is essential for neural crest specification. Development.

[42] Monsoro-Burq AH, Wang E, Harland R (2005). Msx1 and Pax3 cooperate to mediate FGF8 and WNT signals during *Xenopus* neural crest induction. Dev Cell.

[43] Jaurena MB, Juraver-Geslin H, Devotta A, Saint-Jeannet JP (2015). Zic1 controls placode progenitor formation non-cell autonomously by regulating retinoic acid production and transport. Nat Commun.

[44] Woda JM, Pastagia J, Mercola M, Artinger KB (2003). Dlx proteins position the neural plate border and determine adjacent cell fates. Development.

[45] McLarren KW, Litsiou A, Streit A (2003). DLX5 positions the neural crest and preplacode region at the border of the neural plate. Dev Biol.

[46] Rogers CD, Archer TC, Cunningham DD, Grammer TC, Casey EM (2008). Sox3 expression is maintained by FGF signaling and restricted to the neural plate by Vent proteins in the *Xenopus* embryo. Dev Biol.

[47] Buitrago-Delgado E, Nordin K, Rao A, Geary L, LaBonne C (2015). Shared regulatory programs suggest retention of blastula-stage potential in neural crest cells. Science.

[48] Brzezinski JA, Reh TA (2015). Photoreceptor cell fate specification in vertebrates. Development.

[49] Hu M, Krause D, Greaves M, Sharkis S, Dexter M, Heyworth C, Enver T (1997). Multilineage gene expression precedes commitment in the hemopoietic system. Genes Dev.

[50] Nimmo RA, May GE, Enver T (2015). Primed and ready: understanding lineage commitment through single cell analysis. Trends Cell Biol.

[51] Li X, Oghi KA, Zhang J, Krones A, Bush KT, Glass CK, Nigam SK, Aggarwal AK, Maas R, Rose DW (2003). Eya protein phosphatase activity regulates Six1-Dach-Eya transcriptional effects in mammalian organogenesis. Nature.

[52] Riddiford N, Schlosser G. Dissecting the pre-placodal transcriptome to reveal presumptive direct targets of Six1 and Eya1 in cranial placodes. Elife. 2016;5:e17666. 10.7554/eLife.17666. 10.7554/eLife.17666 PMC5035141 27576864

[53] Christophorou NA, Bailey AP, Hanson S, Streit A. Activation of Six1 target genes is required for sensory placode formation. Dev Biol. 2009;336:327–36. 10.1016/j.ydbio.2009.09.025 19781543

[54] Nieuwkoop PD, Faber J (1967). Normal table of *Xenopus laevis* (Daudin).

[55] Sive HL, Grainger RM, Harland RM: Early development of *Xenopus laevis.* Cold Spring Harbor: Cold Spring Harbor Laboratory Press; 2000.

[56] Schlosser G, Awtry T, Brugmann SA, Jensen ED, Neilson K, Ruan G, Stammler A, Voelker D, Yan B, Zhang C (2008). Eya1 and Six1 promote neurogenesis in the cranial placodes in a SoxB1-dependent fashion. Dev Biol.

[57] Sasai N, Mizuseki K, Sasai Y (2001). Requirement of FoxD3-class signaling for neural crest determination in Xenopus. Development.

[58] Penzel R, Oschwald R, Chen Y, Tacke L, Grunz H (1997). Characterization and early embryonic expression of a neural specific transcription factor xSOX3 in *Xenopus laevis*. Int J Dev Biol.

[59] Bang AG, Papalopulu N, Kintner C, Goulding MD (1997). Expression of Pax-3 is initiated in the early neural plate by posteriorizing signals produced by the organizer and by posterior non-axial mesoderm. Development.

[60] Su MW, Suzuki HR, Solursh M, Ramirez F (1991). Progressively restricted expression of a new homeobox-containing gene during *Xenopus laevis* embryogenesis. Development.

[61] Pohl BS, Knöchel W (2002). Temporal and spatial expression patterns of FoxD2 during the early development of Xenopus laevis. Mech Dev.

[62] Walmsley ME, Guille MJ, Bertwistle D, Smith JC, Pizzey JA, Patient RK (1994). Negative control of *Xenopus* GATA-2 by activin and noggin with eventual expression in precursors of the ventral blood islands. Development.

[63] Luo T, Matsuo-Takasaki M, Sargent TD (2001). Distinct roles for distal-less genes Dlx3 and Dlx5 in regulating ectodermal development in *Xenopus*. Mol Reprod Dev.

[64] Zhang C, Basta T, Hernandez-Lagunas L, Simpson P, Stemple DL, Artinger KB, Klymkowsky MW. Repression of nodal expression by maternal B1-type3 SOXs regulates germ layer formation in *Xenopus* and zebrafish. Dev Biol. 2004;273:23–37. 10.1016/j.ydbio.2004.05.019 15302595

[65] Longabaugh WJ, Davidson EH, Bolouri H (2005). Computational representation of developmental genetic regulatory networks. Dev Biol.

[66] Longabaugh WJ, Davidson EH, Bolouri H (2009). Visualization, documentation, analysis, and communication of large-scale gene regulatory networks. Biochim Biophys Acta.

[67] Steventon B, Araya C, Linker C, Kuriyama S, Mayor R (2009). Differential requirements of BMP and Wnt signalling during gastrulation and neurulation define two steps in neural crest induction. Development.

[68] Monsoro-Burq AH, Fletcher RB, Harland RM (2003). Neural crest induction by paraxial mesoderm in *Xenopus* embryos requires FGF signals. Development.

[69] Nichane M, Ren X, Souopgui J, Bellefroid EJ (2008). Hairy2 functions through both DNA-binding and non DNA-binding mechanisms at the neural plate border in *Xenopus*. Dev Biol.

[70] Schlosser G (2006). Induction and specification of cranial placodes. Dev Biol.

