# Supplementary material for: A gene regulatory network underlying the formation of pre-placodal ectoderm in Xenopus laevis
Source: BMC Biol. 2018 Jul 16;16:79. doi: 10.1186/s12915-018-0540-5 (PMC6048776; doi:10.1186/s12915-018-0540-5)
Supplement: Supplementary file 1 — Table S1. Sequences and validation of Morpholinos used. Table S2. Changes in Six1 and Eya1 expression in the non-neural (placodal, epidermal) ectoderm after injection of various MO at high levels. Table S3. Changes in marker gene expression in the non-neural (placodal, epidermal), and neural ectoderm after the injection of various MO. Table S4. Changes in marker gene expression in the non-neural (placodal, epidermal), and neural ectoderm after the injection of various mRNAs for early TFs. Table S5. Changes in marker gene expression in the non-neural (placodal, epidermal), and neural ectoderm after the injection of various mRNAs for hormone-inducible early TFs. Table S6. Changes in marker gene expression in the non-neural (placodal, epidermal), and neural ectoderm after the co-injection of various mRNAs. Table S7. Changes in marker gene expression in the non-neural (placodal, epidermal), and neural ectoderm after the injection of Zic1 and Pax3 mRNAs. Table S8. Changes in marker gene expression in the non-neural (placodal, epidermal), and neural ectoderm after knockdown of Six1 or Eya1. Table S9. Changes in marker gene expression in the non-neural (placodal, epidermal), and neural ectoderm after overexpression of Six1 or Eya1. Table S10. Doses of mRNAs injected. (DOCX 131 kb) [file 12915_2018_540_MOESM1_ESM.docx]

**Table S1** Sequences and validation of Morpholinos used

|  | | |  |  |  |
| --- | --- | --- | --- | --- | --- |
| Morpholino (MO) | Sequence (5’-3’) | MO  Refe-rence | Test of MO efficacy^1^ | Rescue of MO effect on NPB marker | Mouse or zebrafish mutant with deficits in NPB (NC or placode) derivatives |
| Six1 MO1 | GGAAGGCAGCATAGACATGGCTCAG | [[1](#_ENREF_1)] | 1 | FoxD3 | *Six1*: [[2-4](#_ENREF_2)] |
| Six1 MO2 | CGCACACGCAAACACATACACGGG | [[1](#_ENREF_1)] | 1 | FoxD3 | *Six1*: [[2-4](#_ENREF_2)] |
| Eya1 MO1 | TACTATGTGGACTGGTTAGATCCTG | [[5](#_ENREF_5)] | 2 | NeuroD | *Eya1*: [[6-8](#_ENREF_6)] |
| Eya1 MO2 | ATATTTGTTCTGTCAGTGGCAAGTC | [[5](#_ENREF_5)] | 2 | NeuroD | *Eya1*: [[6-8](#_ENREF_6)] |
| Zic1 MO | AAGTCTTCCAACAATGGGCAGCGAA | [[9](#_ENREF_9)] | 3 | FoxD3, Snail2 | *Zic1*: [[10](#_ENREF_10)]; *Zic2*^2^: [[11](#_ENREF_11)] |
| Pax3 MO | TCTCAGTTCCCTTGCCAAGTATTAA | [[12](#_ENREF_12)] | 2 | FoxD3, Snail2 | *Pax3*: [[13](#_ENREF_13), [14](#_ENREF_14)] |
| Hairy2a MO | ATGGTATCTGCGGGCATGTTCAGTT | [[15](#_ENREF_15)] | 1 | Snail2 | -^3^ |
| Hairy2b MO | GGCCATGTTCAGATGTTGTATCCGGA | [[15](#_ENREF_15)] | 1 | Snail2 | -^3^ |
| AP2 MO | GGCGATCCTGCCATTCCCCCATTTT | [[16](#_ENREF_16), [17](#_ENREF_17)] | 2, 4 | Pax3 | *AP2*: [[18](#_ENREF_18), [19](#_ENREF_19)] |
| Msx1 MO | GCCATACAGAGAGATCCGAGCTGAG | [[12](#_ENREF_12)] | 2 | FoxD3, Snail2 | *Msx1*: [[20](#_ENREF_20)] |
| Foxi1a MO | GATCAGCGGCTTCTGCTCTTTCCCA | [[21](#_ENREF_21)] | 3 | FoxD3, Six1, Sox2 | *Foxi1*: [[22](#_ENREF_22), [23](#_ENREF_23)] |
| Vent2 MO | GTCATCTTGTCTGTATTAGTCCT | [[24](#_ENREF_24), [25](#_ENREF_25)] | 4 | Six3 | -^4^ |
| Dlx3 MO | CAGAGCCGGAGAAACGAACCAGACT | [[26](#_ENREF_26)] | 2 | FoxD3, Six1 | *Dlx5*^5^: [[27-29](#_ENREF_27)] |
| ^1^Tests of MO efficacy: 1: Knockdown of protein in vivo; 2: Knockdown of protein after in vitro TNT (Western blot); 3: Knockdown of protein after injection into embryo (Western blot); 4: Saturation of dose response.  ^2^*Zic1*, *Zic2* and *Zic3* have partly redundant functions [[30](#_ENREF_30)].  ^3^*Hes4* *(Hairy2a/2b* ortholog) not found in rodents [[31](#_ENREF_31)].  ^4^*Ventx2* (*Vent2* ortholog) not found in rodents [[25](#_ENREF_25)].  ^5^Early ectodermal expression and function of *Dlx3* in *Xenopus* and zebrafish is mimicked by *Dlx5* in amniotes [[32](#_ENREF_32), [33](#_ENREF_33)]. | | | | | |

**Table S2** Changes in Six1 and Eya1 expression in the non-neural (placodal, epidermal) ectoderm after injection of various MO at high levels­

|  | Injection^1^ | Control MO | *Zic1*  MO | *Pax3*  MO | *Hairy2b*  MO | *AP2*  MO | *Msx1*  MO | *Vent2*  MO | *FoxI1a* MO |
| --- | --- | --- | --- | --- | --- | --- | --- | --- | --- |
| Pheno-type^2^ |  | **%**  **(n)** | **%^3^**  **(n)** | **%^3^**  **(n)** | **%^3^**  **(n)** | **%^3^**  **(n)** | **%^3^**  **(n)** | **%^3^**  **(n)** | **%^3^**  **(n)** |
| *Six1* | **Reduced** | 8^4^  (38) | 50*  (10) | 74**  (47) | 73**  (26) | 67**  (12) | 80**  (27) | 68**  (34) | 64**  (42) |
| *Eya1* | **Reduced** | 4^4^  (45) | 88**  (17) | 41**  (27) | 63**  (24) | 41**  (17) | 67**  (43) | 84**  (19) | 84**  (37) |
| ^1^10-20 ng injected  ^2^% of embryos affected at stage 14-16  ^3^Significant differences (Two-tailed Fisher’s exact test; *: p< 0.05, **: p< 0.001) to control MO injections are indicated  ^4^Same data as in Table S9  n: number of embryos analyzed | | | | | | | | | |

**­­**

**Table S3** Changes in marker gene expression in the non-neural (placodal, epidermal), and neural ectoderm after the injection of various MO.­

|  |  | Injection^1^ | Control MO | *Zic1*  MO | *Pax3*  MO | *Hairy2b*  MO | *AP2*  MO | *Msx1*  MO | *Vent2*  MO | *FoxI1a* MO |
| --- | --- | --- | --- | --- | --- | --- | --- | --- | --- | --- |
| Pheno-type^2^ |  |  | **%**  **(n)** | **%^3^**  **(n)** | **%^3^**  **(n)** | **%^3^**  **(n)** | **%^3^**  **(n)** | **%^3^**  **(n)** | **%^3^**  **(n)** | **%^3^**  **(n)** |
| *Six1* | **Non-neural** | **Reduced** | 13  (31) | 72**  (32) | 47*  (36) | 57**  (42) | 64**  (47) | 26  (89) | 56**  (43) | 45*  (40) |
|  |  | **Increased /Ectopic** | 0  (31) | 0  (32) | 0  (36) | 2  (42) | 6  (47) | 0  (89) | 9  (43) | 22*  (40) |
|  | **Neural** | **Reduced** | 0  (31) | 0  (32) | 0  (36) | 0  (42) | 0  (47) | 0  (89) | 0  (43) | 0  (40) |
|  |  | **Increased /Ectopic** | 0  (31) | 0  (32) | 0  (36) | 0  (42) | 0  (47) | 0  (89) | 0  (43) | 0  (40) |
|  |  | **Lateral displacement** | 0  (31) | 37**  (30) | 41**  (27 | 10  (29) | 0  (47) | 62**  (89) | 32*  (22) | 32*  (19) |
| *Eya1* | **Non-neural** | **Reduced** | 3  (29) | 53**  (19) | 39*  (13) | 62**  (42) | 57**  (40) | 27*  (78) | 63**  (30) | 62**  (34) |
|  |  | **Increased /Ectopic** | 0  (29) | 10  (19) | 0  (13) | 0  (42) | 5  (40) | 0  (78) | 0  (30) | 0  (34) |
|  | **Neural** | **Reduced** | 0  (29) | 0  (19) | 0  (13) | 0  (42) | 0  (40) | 0  (78) | 0  (30) | 0  (34) |
|  |  | **Increased /Ectopic** | 0  (29) | 0  (19) | 0  (13) | 0  (42) | 0  (40) | 0  (78) | 0  (30) | 0  (34) |
|  |  | **Lateral displacement** | 0  (29) | 55**  (22) | 29*  (7) | 25*  (42) | 0  (40) | 41**  (32) | 47**  (17) | 35**  (23) |
| *FoxD3* | **Non-neural** | **Reduced** | 0  (31) | 0  (30) | 0  (28) | 0  (37) | 0  (49) | 0  (60) | 0  (27) | 0  (30) |
|  |  | **Increased /Ectopic** | 0  (31) | 0  (30) | 0  (28) | 0  (37) | 0  (49) | 0  (60) | 0  (27) | 0  (30) |
|  | **Neural** | **Reduced** | 3  (31) | 80**  (30) | 57**  (28) | 19  (37) | 39**  (49) | 75**  (60) | 67**  (27) | 20  (30) |
|  |  | **Increased /Ectopic** | 0  (31) | 0  (30) | 7  (28) | 38**  (37) | 27*  (49) | 0  (60) | 11  (27) | 30**  (30) |
|  |  | **Lateral displacement** | 0  (31) | 41**  (27) | 54**  (28) | 33**  (21) | 0  (49) | 63**  (32) | 31*  (27) | 19*  (21) |
| *Sox3* | **Non-neural** | **Reduced** | 2  (50) | 44**  (18) | 0  (17) | 21*  (29) | 64**  (47) | 17*  (70) | 46**  (28) | 35**  (26) |
|  |  | **Increased /Ectopic** | 0  (50) | 0  (18) | 47**  (17) | 17*  (29) | 0  (47) | 83**  (70) | 4  (28) | 15*  (26) |
|  | **Neural** | **Reduced** | 0  (50) | 0  (18) | 0  (17) | 0  (29) | 2  (47) | 0  (70) | 0  (28) | 0  (26) |
|  |  | **Increased /Ectopic** | 0  (50) | 0  (18) | 0  (17) | 0  (29) | 0  (47) | 0  (70) | 0  (28) | 0  (26) |
|  |  | **Lateral displacement** | 0  (50) | 48**  (29) | 53**  (21) | 38**  (29) | 0  (47) | 76**  (80) | 62**  (29) | 20*  (15) |
| ^1^1 ng injected  ^2^% of embryos affected at stage 14-16  ^3^Significant differences (Two-tailed Fisher’s exact test; *: p< 0.05, **: p< 0.001) to control MO injections are indicated  n: number of embryos analyzed; nd: not determined | | | | | | | | | | |

**­­**

**Table S4** Changes in marker gene expression in the non-neural (placodal, epidermal), and neural ectoderm after the injection of various mRNAs­ for early TFs

|  |  | Injection^1^ | *Zic1*  mRNA | *Pax3*  mRNA | *Hairy2b*  mRNA | *AP2*  mRNA | *Msx1*  mRNA | *Vent2*  mRNA | *FoxI1a*  mRNA |
| --- | --- | --- | --- | --- | --- | --- | --- | --- | --- |
| Pheno-type^2^ |  |  | **%**  **(n)** | **%**  **(n)** | **%**  **(n)** | **%**  **(n)** | **%**  **(n)** | **%**  **(n)** | **%**  **(n)** |
| *Six1* | **Non-neural** | **Reduced** | 60  (104) | 49  (35) | 40  (20) | 74  (54) | 52  (50) | 72  (29) | 39  (18) |
|  |  | **Increased /Ectopic** | 10  (104) | 0  (35) | 5  (20) | 11  (54) | 32  (50) | 0  (29) | 11  (18) |
|  | **Neural** | **Reduced** | 0  (104) | 0  (35) | 0  (20) | 0  (62) | 0  (50) | 0  (29) | 0  (18) |
|  |  | **Increased /Ectopic** | 0  (104) | 0  (35) | 40  (20) | 43  (62) | 32  (50) | 55  (29) | 0  (18) |
| *Eya1* | **Non-neural** | **Reduced** | 68  (76) | 42  (45) | 100  (1) | 62  (69) | 53  (30) | 76  (29) | 26  (23) |
|  |  | **Increased /Ectopic** | 3  (76) | 0  (45) | 0  (1) | 3  (69) | 7  (30) | 0  (29) | 22  (23) |
|  | **Neural** | **Reduced** | 0  (76) | 0  (45) | 0  (1) | 0  (69) | 0  (30) | 0  (29) | 0  (23) |
|  |  | **Increased /Ectopic** | 0  (76) | 0  (45) | 0  (1) | 6  (69) | 3  (30) | 48*  (29) | 0  (23) |
| *FoxD3* | **Non-neural** | **Reduced** | 0  (57) | 0  (84) | nd | 0  (30) | 0  (14) | 0  (77) | 0  (15) |
|  |  | **Increased /Ectopic** | 0  (57) | 0  (81) | nd | 0  (30) | 0  (14) | 0  (23) | 0  (24) |
|  | **Neural** | **Reduced** | 42  (57) | 31  (84) | nd | 20  (30) | 29  (14) | 74  (77) | 17  (24) |
|  |  | **Increased /Ectopic** | 35  (57) | 54  (81) | nd | 60  (30) | 57  (14) | 10  (77) | 50  (24) |
| *Sox3* | **Non-neural** | **Reduced** | 37  (52) | 73  (64) | 54  (11) | 58  (26) | 64  (11) | 69  (55) | 18  (11) |
|  |  | **Increased /Ectopic** | 23  (52) | 50  (64) | 0  (11) | 19  (26) | 0  (11) | 69  (55) | 0  (11) |
|  | **Neural** | **Reduced** | 21  (52) | 28  (46) | 71  (7) | 60  (45) | 42  (19) | 60  (25) | 0  (11) |
|  |  | **Increased /Ectopic** | 0  (52) | 0  (46) | 0  (11) | 0  (45) | 0  (19) | 0  (25) | 0  (11) |
| ^1^See Table S10 for amounts injected  ^2^% of embryos affected at stage 14-16  n: number of embryos analyzed; nd: not determined  ^3^Not analyzable due to strong gastrulation defects | | | | | | | | | |

**Table S5** Changes in marker gene expression in the non-neural (placodal, epidermal), and neural ectoderm after the injection of various mRNAs­ for hormone-inducible early TFs

|  |  | Injection^1^ | *Zic1* -GR  mRNA | *Pax3-GR*  mRNA | *Hairy2-GR*  mRNA | *AP2-GR*  mRNA | *Msx1-GR*  mRNA | *Vent2-GR*  mRNA | *FoxI1a-GR*  mRNA |
| --- | --- | --- | --- | --- | --- | --- | --- | --- | --- |
| Pheno-type^2^ |  |  | **%**  **(n)** | **%**  **(n)** | **%**  **(n)** | **%**  **(n)** | **%**  **(n)** | **%**  **(n)** | **%**  **(n)** |
| *Six1* | **Non-neural** | **Reduced** | 9  (22) | 100  (17) | 76  (37) | 72  (43) | 15  (20) | 79  (19) | 20  (41) |
|  |  | **Increased /Ectopic** | 0  (22) | 0  (17) | 8  (37) | 44  (18) | 60  (20) | 0  (19) | 0  (41) |
|  | **Neural** | **Reduced** | 0  (22) | 0  (17) | 0  (37) | 0  (41) | 0  (20) | 0  (19) | 0  (41) |
|  |  | **Increased /Ectopic** | 0  (22) | 0  (17) | 0  (37) | 27  (41) | 60  (20) | 5  (19) | 0  (41) |
| *Eya1* | **Non-neural** | **Reduced** | 31  (13) | 83  (18) | 50  (22) | 73  (37) | 8  (13) | 68  (25) | 23  (31) |
|  |  | **Increased /Ectopic** | 0  (13) | 0  (18) | 0  (22) | 19  (37) | 54  (13) | 0  (25) | 0  (31) |
|  | **Neural** | **Reduced** | 0  (13) | 0  (18) | 0  (22) | 0  (19) | 0  (13) | 0  (25) | 0  (31) |
|  |  | **Increased /Ectopic** | 0  (13) | 5  (18) | 0  (22) | 19  (37) | 45  (11) | 0  (25) | 0  (31) |
| *FoxD3* | **Non-neural** | **Reduced** | 0  (12) | 0  (16) | 0  (31) | 0  (24) | 0  (27) | 0  (34) | 0  (25) |
|  |  | **Increased /Ectopic** | 0  (12) | 0  (16) | 0  (31) | 0  (24) | 0  (27) | 0  (34) | 0  (25) |
|  | **Neural** | **Reduced** | 16  (12) | 31  (16) | 68  (31) | 28  (24) | 19  (27) | 67  (34) | 28  (25) |
|  |  | **Increased /Ectopic** | 75  (12) | 31  (16) | 0  (31) | 33  (24) | 78  (27) | 23  (34) | 0  (25) |
| *Sox3* | **Non-neural** | **Reduced** | 0  (32) | 50  (26) | 60  (26) | 52  (17) | 0  (12) | 75  (12) | 0  (24) |
|  |  | **Increased /Ectopic** | 0  (32) | 0  (26) | 35  (26) | 0  (17) | 0  (12) | 0  (12) | 0  (24) |
|  | **Neural** | **Reduced** | 0  (32) | 50  (26) | 0  (26) | 0  (17) | 33  (12) | 56  (16) | 0  (24) |
|  |  | **Increased /Ectopic** | 0  (32) | 0  (26) | 0  (26) | 0  (17) | 0  (12) | 0  (14) | 0  (24) |
| ^1^See Table S10 for amounts injected  ^2^% of embryos affected at stage 14-16  n: number of embryos analyzed; nd: not determined | | | | | | | | | |

**Table S6** Changes in marker gene expression in the non-neural (placodal, epidermal), and neural ectoderm after the co-injection of various mRNAs­

|  |  | Injection^1^ | *AP2*+*Zic1* mRNA | *AP2*+*Sox3* mRNA | *AP2* +*Pax3* mRNA | *Dlx3* +*Zic1* mRNA | *Dlx3*+*Sox3* mRNA | *Dlx3*+*Pax3* mRNA | *Dlx3* mRNA | *Sox3*  mRNA | *Dlx3* MO +*Zic1* mRNA | *Msx1+Sox3* mRNA |
| --- | --- | --- | --- | --- | --- | --- | --- | --- | --- | --- | --- | --- |
| Pheno-type^2^ |  |  | **%**  **(n)** | **%**  **(n)** | **%**  **(n)** | **%**  **(n)** | **%**  **(n)** | **%**  **(n)** | **%**  **(n)** | **%**  **(n)** | **%**  **(n)** | **%**  **(n)** |
| *Six1* | **Non-neural** | **Reduced** | 49  (37) | 68  (63) | 63  (24) | 40  (47) | 92  (13) | 69  (13) | 65  (43) | 75  (26) | 37  (52) | 72  (39) |
|  |  | **Increased /Ectopic** | 8  (37) | 0  (63) | 17  (24) | 32  (47) | 0  (13) | 25  (20) | 0  (43) | 0  (26) | 0  (52) | 0  (39) |
|  | **Neural** | **Reduced** | 0  (37) | 0  (63) | 0  (24) | 0  (47) | 0  (13) | 0  (13) | 0  (38) | 0  (26) | 0  (52) | 0  (39) |
|  |  | **Increased /Ectopic** | 11  (37) | 33  (64) | 38  (24) | 36  (47) | 0  (13) | 69  (13) | 50  (38) | 0  (26) | 0  (52) | 5  (39) |
| *Eya1* | **Non-neural** | **Reduced** | 60  (20) | 48  (44) | 42  (19) | 32  (59) | 100  (6) | 70  (20) | 74  (46) | 69  (16) | 57  (35) | 71  (38) |
|  |  | **Increased /Ectopic** | 0  (20) | 0  (44) | 0  (19) | 36  (59) | 0  (6) | 30  (20) | 0  (46) | 0  (16) | 0  (35) | 0  (38) |
|  | **Neural** | **Reduced** | 0  (20) | 0  (44) | 0  (19) | 0  (59) | 0  (6) | 0  (14) | 0  (46) | 0  (16) | 0  (35) | 0  (38) |
|  |  | **Increased /Ectopic** | 5  (20) | 11  (44) | 16  (19) | 14  (59) | 0  (13) | 21  (14) | 14  (43) | 0  (16) | 0  (35) | 5  (38) |
| *FoxD3* | **Non-neural** | **Reduced** | nd | nd | nd | 0  (55) | nd | 0  (36) | 0  (17) | nd | 0  (41) | nd |
|  |  | **Increased /Ectopic** | nd | nd | nd | 0  (55) | nd | 0  (36) | 0  (17) | nd | 0  (41) | nd |
|  | **Neural** | **Reduced** | nd | nd | nd | 35  (55) | nd | 72  (36) | 82  (17) | nd | 15  (41) | nd |
|  |  | **Increased /Ectopic** | nd | nd | nd | 40  (55) | nd | 17  (36) | 0  (17) | nd | 5  (41) | nd |
| *Sox3* | **Non-neural** | **Reduced** | nd | 0  (45) | nd | 24  (55) | 0  (52) | 26  (19) | 13  (16) | 0  (29) | 34  (38) | nd |
|  |  | **Increased /Ectopic** | nd | 89  (45) | nd | 44  (55) | 87  (52) | 89  (19) | 17  (12) | 100  (29) | 0  (38) | nd |
|  | **Neural** | **Reduced** | nd | 0  (45) | nd | 4  (55) | 0  (52) | 15  (19) | 19  (18) | 0  (29) | 0  (38) | nd |
|  |  | **Increased /Ectopic** | nd | 0  (45) | nd | 0  (55) | 0  (52) | 0  (19) | 0  (18) | 0  (29) | 0  (38) | nd |
| ^1^See Table S10 for amounts injected  ^2^% of embryos affected at stage 14-16  n: number of embryos analyzed; nd: not determined | | | | | | | | | | | | |

**Table S7** Changes in marker gene expression in the non-neural (placodal, epidermal), and neural ectoderm after the injection of *Zic1* and *Pax3* mRNAs­

|  |  | Injection^1^ | *Zic1*  mRNA | *Pax3*  mRNA |
| --- | --- | --- | --- | --- |
| Pheno-type^2^ |  |  | **%**  **(n)** | **%**  **(n)** |
| *FoxI1* | **Non-neural** | **Reduced** | 40  (15) | 44  (18) |
|  |  | **Increased /Ectopic** | 7  (15) | 6  (18) |
|  | **Neural** | **Reduced** | 0  (15) | 0  (18) |
|  |  | **Increased /Ectopic** | 0  (15) | 0  (18) |
| *Dlx3* | **Non-neural** | **Reduced** | 0  (15) | 0  (21) |
|  |  | **Increased /Ectopic** | 0  (15) | 0  (21) |
|  | **Neural** | **Reduced** | 0  (15) | 0  (21) |
|  |  | **Increased /Ectopic** | 0  (15) | 0  (21) |
| *GATA2* | **Non-neural** | **Reduced** | 42  (19) | 33  (18) |
|  |  | **Increased /Ectopic** | 11  (19) | 0  (18) |
|  | **Neural** | **Reduced** | 0  (19) | 0  (18) |
|  |  | **Increased /Ectopic** | 0  (19) | 0  (18) |
| ^1^See Table S10 for amounts injected  ^2^% of embryos affected at stage 14-16  n: number of embryos analyzed | | | | |

**Table S8** Changes in marker gene expression in the non-neural (placodal, epidermal), and neural ectoderm after knockdown of Six1 or Eya1

|  |  | Injection^1^ | Control MO^2^ | *Six1*  MO1+  MO2^1^ | *Eya1*  MO1+  MO2^1^ |
| --- | --- | --- | --- | --- | --- |
| Pheno-type^3^ |  |  | **%**  **(n)** | **%^4^**  **(n)** | **%^4^**  **(n)** |
| *Six1* | **Non-neural** | **Reduced** | 8  (38) | nd | 48**  (99) |
|  |  | **Increased /Ectopic** | 0  (38) | nd | 11  (18) |
|  | **Neural** | **Reduced** | 0  (38) | nd | 0  (99) |
|  |  | **Increased /Ectopic** | 0  (38) | nd | 0  (18) |
|  |  | **Lateral displacement** | 5  (38) | nd | 61**  (67) |
| *Eya1* | **Non-neural** | **Reduced** | 4  (45) | 56**  (16) | 54**  (39) |
|  |  | **Increased /Ectopic** | 0  (45) | 0  (16) | 15*  (39) |
|  | **Neural** | **Reduced** | 0  (45) | 0  (16) | 0  (39) |
|  |  | **Increased /Ectopic** | 0  (45) | 0  (16) | 0  (39) |
|  |  | **Lateral displacement** | 0  (34) | 64**  (22) | 61*  (41) |
| *FoxD3* | **Non-neural** | **Reduced** | 0  (21) | 0  (38) | 0  (87) |
|  |  | **Increased /Ectopic** | 0  (21) | 0  (38) | 0  (87) |
|  | **Neural** | **Reduced** | 5  (21) | 60**  (38) | 58**  (87) |
|  |  | **Increased /Ectopic** | 0  (21) | 0  (38) | 0  (87) |
|  |  | **Lateral displacement** | 14  (21) | 70*  (10) | 55**  (62) |
| *Sox3* | **Non-neural** | **Reduced** | 12  (60) | 62**  (153) | 30*  (156) |
|  |  | **Increased /Ectopic** | 0  (60) | 0  (153) | 12*  (156) |
|  | **Neural** | **Reduced** | 4  (25) | 0  (153) | 0  (156) |
|  |  | **Increased /Ectopic** | 0  (25) | 0  (153) | 0  (156) |
|  |  | **Lateral displacement** | 7  (24) | 70**  (20) | 82**  (136) |
| *Zic1* | **Non-neural** | **Reduced** | 0  (20) | 0  (48) | 0  (67) |
|  |  | **Increased /Ectopic** | 0  (20) | 0  (48) | 0  (67) |
|  | **Neural** | **Reduced** | 0  (20) | 15  (48) | 0  (67) |
|  |  | **Increased /Ectopic** | 0  (20) | 58**  (48) | 75**  (67) |
|  |  | **Lateral displacement** | 15  (20) | 40  (48) | 54*  (67) |
| *Pax3* | **Non-neural** | **Reduced** | 18  (28) | 0  (46) | 3  (31) |
|  |  | **Increased /Ectopic** | 0  (28) | 4  (46) | 6  (31) |
|  | **Neural** | **Reduced** | 18  (28) | 0^5^  (46) | 13  (31) |
|  |  | **Increased /Ectopic** | 0  (28) | 35**  (46) | 23*  (31) |
|  |  | **Lateral displacement** | 0  (28) | 41**  (46) | 32**  (31) |
| *AP2* | **Non-neural** | **Reduced** | 0  (18) | 0  (50) | 0  (41) |
|  |  | **Increased /Ectopic** | 0  (18) | 40**  (50) | 0  (41) |
|  | **Neural** | **Reduced** | 17  (18) | 28  (43) | 37  (41) |
|  |  | **Increased /Ectopic** | 0  (18) | 72**  (50) | 27*  (41) |
|  |  | **Lateral displacement** | 17  (18) | 44*  (43) | 44  (41) |
| *Msx1* | **Non-neural** | **Reduced** | 8  (25) | 0  (36) | 29  (45) |
|  |  | **Increased /Ectopic** | 0  (25) | 47**  (36) | 46**  (37) |
|  | **Neural** | **Reduced** | 0  (25) | 25*  (36) | 22*  (45) |
|  |  | **Increased /Ectopic** | 0  (25) | 50**  (36) | 31*  (37) |
|  |  | **Lateral displacement** | 0  (25) | 36*  (11) | 48**  (55) |
| *FoxI1a* | **Non-neural** | **Reduced** | 15  (20) | 39  (41) | 40  (78) |
|  |  | **Increased /Ectopic** | 0  (20) | 34*  (41) | 13  (78) |
|  | **Neural** | **Reduced** | 0  (20) | 0  (41) | 0  (78) |
|  |  | **Increased /Ectopic** | 0  (20) | 0  (41) | 1  (78) |
|  |  | **Lateral displacement** | 0  (20) | 29*  (41) | 45**  (73) |
| *Dlx3* | **Non-neural** | **Reduced** | 0  (32) | 40**  (30) | 61**  (46) |
|  |  | **Increased /Ectopic** | 0  (32) | 13*  (30) | 0  (46) |
|  | **Neural** | **Reduced** | 0  (32) | 0  (30) | 0  (46) |
|  |  | **Increased /Ectopic** | 0  (32) | 0  (30) | 0  (46) |
|  |  | **Lateral displacement** | 0  (32) | 50**  (30) | 55**  (37) |
| *GATA2* | **Non-neural** | **Reduced** | 0  (25) | 28*  (39) | 21*  (29) |
|  |  | **Increased /Ectopic** | 0  (25) | 5  (39) | 7  (29) |
|  | **Neural** | **Reduced** | 0  (25) | 0  (39) | 0  (29) |
|  |  | **Increased /Ectopic** | 0  (25) | 0  (39) | 0  (29) |
|  |  | **Lateral displacement** | 4  (25) | 54**  (39) | 35*  (29) |
| ^1^10-20 ng injected  ^2^Except for AP2, data from [[26](#_ENREF_26)].  ^3^% of embryos affected at stage 14-16  ^4^Significant differences (Two-tailed Fisher’s exact test; *: p< 0.05, **: p< 0.001) to control MO injections are indicated  n: number of embryos analyzed; nd: not determined | | | | | |

**Table S9** Changes in marker gene expression in the non-neural (placodal, epidermal), and neural ectoderm after overexpression of Six1 or Eya1

|  |  | Injection^1^ | *Six1*  mRNA | *Eya1*  mRNA | *Six1+*  *Eya1*  mRNA |
| --- | --- | --- | --- | --- | --- |
| Pheno-type^2^ |  |  | **%**  **(n)** | **%**  **(n)** | **%**  **(n)** |
| *Six1* | **Non-neural** | **Reduced** | nd | 13  (16) | nd |
|  |  | **Increased /Ectopic** | nd | 50  (16) | nd |
|  | **Neural** | **Reduced** | nd | 0  (16) | nd |
|  |  | **Increased /Ectopic** | nd | 19  (16) | nd |
| *Eya1* | **Non-neural** | **Reduced** | 89  (35) | nd | nd |
|  |  | **Increased /Ectopic** | 0  (35) | nd | nd |
|  | **Neural** | **Reduced** | 0  (35) | nd | nd |
|  |  | **Increased /Ectopic** | 0  (35) | nd | nd |
| *FoxD3* | **Non-neural** | **Reduced** | 0  (67) | 0  (115) | 0  (13) |
|  |  | **Increased /Ectopic** | 0  (67) | 0  (115) | (13) |
|  | **Neural** | **Reduced** | 77  (67) | 5  (115) | 80  (13) |
|  |  | **Increased /Ectopic** | 0  (67) | 37  (115) | 23  (13) |
| *Sox3* | **Non-neural** | **Reduced** | 55  (57) | 21  (19) | nd |
|  |  | **Increased /Ectopic** | 7  (57) | 21  (19) | nd |
|  | **Neural** | **Reduced** | 38  (66) | 37  (62) | nd |
|  |  | **Increased /Ectopic** | 0  (66) | 0  (62) | nd |
| *Zic1* | **Non-neural** | **Reduced** | 0  (24) | 0  (41) | 0  (25) |
|  |  | **Increased /Ectopic** | 0  (24) | 0  (41) | 0  (25) |
|  | **Neural** | **Reduced** | 0  (24) | 0  (41) | 80  (25) |
|  |  | **Increased /Ectopic** | 92  (24) | 81  (41) | 0  (25) |
| *Pax3* | **Non-neural** | **Reduced** | 44  (27) | 21  (24) | nd |
|  |  | **Increased /Ectopic** | 15  (20) | 0  (24) | nd |
|  | **Neural** | **Reduced** | 50  (34) | 0  (24) | nd |
|  |  | **Increased /Ectopic** | 35  (34) | 17  (24) | nd |
| *AP2* | **Non-neural** | **Reduced** | 11  (36) | 0  (23) | nd |
|  |  | **Increased /Ectopic** | 14  (36) | 0  (23) | nd |
|  | **Neural** | **Reduced** | 11  (36) | 0  (23) | nd |
|  |  | **Increased /Ectopic** | 33  (36) | 48  (23) | nd |
| *Msx1* | **Non-neural** | **Reduced** | 27  (30) | 60  (43) | nd |
|  |  | **Increased /Ectopic** | 73  (30) | 0  (43) | nd |
|  | **Neural** | **Reduced** | 0  (30) | 60  (43) | nd |
|  |  | **Increased /Ectopic** | 77  (30) | 0  (43) | nd |
| *FoxI1a* | **Non-neural** | **Reduced** | 47  (19) | 14  (42) | nd |
|  |  | **Increased /Ectopic** | 21  (19) | 29  (42) | nd |
|  | **Neural** | **Reduced** | 0  (19) | 0  (42) | nd |
|  |  | **Increased /Ectopic** | 0  (19) | 3  (32) | nd |
| *Dlx3* | **Non-neural** | **Reduced** | 68  (19) | 70  (43) | nd |
|  |  | **Increased /Ectopic** | 0  (19) | 0  (43) | nd |
|  | **Neural** | **Reduced** | 0  (19) | 0  (43) | nd |
|  |  | **Increased /Ectopic** | 0  (19) | 0  (43) | nd |
| *GATA2* | **Non-neural** | **Reduced** | 36  (14) | 0  (20) | nd |
|  |  | **Increased /Ectopic** | 0  (14) | 0  (20) | nd |
|  | **Neural** | **Reduced** | 0  (14) | 0  (20) | nd |
|  |  | **Increased /Ectopic** | 0  (14) | 0  (20) | nd |
| ^1^ See Table S10 for amounts of mRNA injected  ^2^% of embryos affected at stage 14-16  n: number of embryos analyzed; nd: not determined | | | | | |

**Table S10** Doses of mRNAs injected

| **Gene name** | **Plasmid** | **Injected dose** | **Reference** |
| --- | --- | --- | --- |
| ***Six1*** | **pDH105-Six1** | **500 pg** | [[34](#_ENREF_34)] |
| ***EnRSix1*** | **pCS2^+^-EnRSix1** | **500 pg** | [[1](#_ENREF_1)] |
| ***Eya1*** | **pCS2^+^-Eya1α** | **500 pg** | [[35](#_ENREF_35)] |
| ***GR-Eya1*** | **pCS2^+^-GR-myc-Eya1α** | **500 pg** | [[5](#_ENREF_5)] |
| ***GR-Six1*** | **pCS2^+^-GR-myc-Six1** | **500 pg** | [[5](#_ENREF_5)] |
| ***Sox3*** | **pCS2^+^-Sox3-V5His** | **500 pg** | [[36](#_ENREF_36)] |
| ***Zic1*** | **pCS2^+^- Zic1** | **250 pg** | [[37](#_ENREF_37)] |
| ***GR-Zic1*** | **pCS2^+^- Zic1-GR** | **500 pg** | New construct |
| ***Pax3*** | **pCS108-XL-Pax3** | **125 pg** | [[12](#_ENREF_12)] |
| ***GR-Pax3*** | **pCS2^+^-Pax3-GR** | **500 pg** | [[38](#_ENREF_38)] |
| ***Hairy2b*** | **pCS2^+^-Flag-xHairy2b** | **250-500 pg** | [[15](#_ENREF_15)] |
| ***GR-Hairy2b*** | **pCS2^+^-Hairy2b-MT-GR** | **500 pg** | [[15](#_ENREF_15)] |
| ***AP2*** | **pCS108-XL-AP2 alpha** | **250 pg** | [[17](#_ENREF_17)] |
| ***GR-AP2*** | **pCS2^+^-GR-XAP2** | **500 pg** | [[39](#_ENREF_39)] |
| ***Msx1*** | **pSP64TXB-xMsx1** | **250 pg** | [[40](#_ENREF_40)] |
| ***GR-Msx1*** | **pCS2^+^-Msx1-GR** | **500 pg** | [[41](#_ENREF_41)] |
| ***Foxi1a*** | **pCS2^+^- xFoxi1a** | **50 pg** | [[21](#_ENREF_21)] |
| ***GR-Foxi1a*** | **pCS2^+^- Foxi1a-GR** | **500 pg** | New construct |
| ***Vent2*** | **pCS2^+^-MT-Xvent2** | **500 pg** | [[42](#_ENREF_42)] |
| ***GR-Vent2*** | **pT7Ts-GR-HA-Xvent2** | **500 pg** | [[42](#_ENREF_42)] |
| ***Dlx3*** | **pCS2^+^-EBMyc-Dlx3** | **50-100 pg** | [[26](#_ENREF_26)]; kindly provided by Dr T. Sargent, NICHD, Rockville, MD, USA |

­

**References**

1. Brugmann SA, Pandur PD, Kenyon KL, Pignoni F, Moody SA: **Six1 promotes a placodal fate within the lateral neurogenic ectoderm by functioning as both a transcriptional activator and repressor**. *Development* 2004, **131**:5871-5881.

2. Laclef C, Souil E, Demignon J, Maire P: **Thymus, kidney and craniofacial abnormalities in Six1 deficient mice**. *MechDev* 2003, **120**(6):669-679.

3. Zheng W, Huang L, Wei ZB, Silvius D, Tang B, Xu PX: **The role of Six1 in mammalian auditory system development**. *Development* 2003, **130**(17):3989-4000.

4. Li X, Oghi KA, Zhang J, Krones A, Bush KT, Glass CK, Nigam SK, Aggarwal AK, Maas R, Rose DW *et al*: **Eya protein phosphatase activity regulates Six1-Dach-Eya transcriptional effects in mammalian organogenesis**. *Nature* 2003, **426**(6964):247-254.

5. Schlosser G, Awtry T, Brugmann SA, Jensen ED, Neilson K, Ruan G, Stammler A, Voelker D, Yan B, Zhang C *et al*: **Eya1 and Six1 promote neurogenesis in the cranial placodes in a SoxB1-dependent fashion**. *DevBiol* 2008, **320**:199-214.

6. Xu PX, Adams J, Peters H, Brown MC, Heaney S, Maas R: **Eya1-deficient mice lack ears and kidneys and show abnormal apoptosis of organ primordia**. *Nature Genet* 1999, **23**(1):113-117.

7. Zou D, Silvius D, Fritzsch B, Xu PX: **Eya1 and Six1 are essential for early steps of sensory neurogenesis in mammalian cranial placodes**. *Development* 2004, **131**(22):5561-5572.

8. Kozlowski DJ, Whitfield TT, Hukriede NA, Lam WK, Weinberg ES: **The zebrafish dog-eared mutation disrupts eya1, a gene required for cell survival and differentiation in the inner ear and lateral line**. *Dev Biol* 2005, **277**(1):27-41.

9. Sato T, Sasai N, Sasai Y: **Neural crest determination by co-activation of Pax3 and Zic1 genes in Xenopus ectoderm**. *Development* 2005, **132**(10):2355-2363.

10. Aruga J, Mizugishi K, Koseki H, Imai K, Balling R, Noda T, Mikoshiba K: **Zic1 regulates the patterning of vertebral arches in cooperation with Gli3**. *Mech Dev* 1999, **89**(1-2):141-150.

11. Elms P, Siggers P, Napper D, Greenfield A, Arkell R: **Zic2 is required for neural crest formation and hindbrain patterning during mouse development**. *Dev Biol* 2003, **264**(2):391-406.

12. Monsoro-Burq AH, Wang E, Harland R: **Msx1 and Pax3 cooperate to mediate FGF8 and WNT signals during Xenopus neural crest induction**. *DevCell* 2005, **8**(2):167-178.

13. Epstein DJ, Vekemans M, Gros P: **Splotch (Sp2H), a mutation affecting development of the mouse neural tube, shows a deletion within the paired homeodomain of Pax-3**. *Cell* 1991, **67**(4):767-774.

14. Boudjadi S, Chatterjee B, Sun W, Vemu P, Barr FG: **The expression and function of PAX3 in development and disease**. *Gene* 2018.

15. Nichane M, de Croze N, Ren X, Souopgui J, Monsoro-Burq AH, Bellefroid EJ: **Hairy2-Id3 interactions play an essential role in Xenopus neural crest progenitor specification**. *DevBiol* 2008, **15**:355-367.

16. Luo T, Lee YH, Saint-Jeannet JP, Sargent TD: **Induction of neural crest in Xenopus by transcription factor AP2alpha**. *ProcNatlAcadSciUSA* 2003, **100**(2):532-537.

17. de Croze N, Maczkowiak F, Monsoro-Burq AH: **Reiterative AP2a activity controls sequential steps in the neural crest gene regulatory network**. *ProcNatlAcadSciUSA* 2011, **108**(1):155-160.

18. Schorle H, Meier P, Buchert M, Jaenisch R, Mitchell PJ: **Transcription factor AP-2 essential for cranial closure and craniofacial development**. *Nature* 1996, **381**:235-238.

19. Zhang J, Hagopian-Donaldson S, Serbedzija G, Elsemore J, Plehn-Dujowich D, McMahon AP, Flavell RA, Williams T: **Neural tube, skeletal and body wall defects in mice lacking transcription factor AP-2**. *Nature* 1996, **381**(6579):238-241.

20. Satokata I, Maas R: **Msx1 deficient mice exhibit cleft palate and abnormalities of craniofacial and tooth development**. *NatGenet* 1994, **6**(4):348-356.

21. Matsuo-Takasaki M, Matsumura M, Sasai Y: **An essential role of Xenopus Foxi1a for ventral specification of the cephalic ectoderm during gastrulation**. *Development* 2005, **132**(17):3885-3894.

22. Hulander M, Wurst W, Carlsson P, Enerback S: **The winged helix transcription factor Fkh10 is required for normal development of the inner ear**. *Nature Genet* 1998, **20**(4):374-376.

23. Solomon KS, Kudoh T, Dawid IB, Fritz A: **Zebrafish foxi1 mediates otic placode formation and jaw development**. *Development* 2003, **130**(5):929-940.

24. Sander V, Reversade B, De Robertis EM: **The opposing homeobox genes Goosecoid and Vent1/2 self-regulate Xenopus patterning**. *Embo J* 2007, **26**(12):2955-2965.

25. Scerbo P, Girardot F, Vivien C, Markov GV, Luxardi G, Demeneix B, Kodjabachian L, Coen L: **Ventx factors function as nanog-like guardians of developmental potential in Xenopus**. *PLoSONE* 2012, **7**(5):e36855.

26. Pieper M, Ahrens K, Rink E, Peter A, Schlosser G: **Differential distribution of competence for panplacodal and neural crest induction to non-neural and neural ectoderm**. *Development* 2012, **139**(6):1175-1187.

27. Acampora D, Merlo GR, Paleari L, Zerega B, Postiglione MP, Mantero S, Bober E, Barbieri O, Simeone A, Levi G: **Craniofacial, vestibular and bone defects in mice lacking the Distal-less-related gene Dlx5**. *Development* 1999, **126**(17):3795-3809.

28. Depew MJ, Liu JK, Long JE, Presley R, Meneses JJ, Pedersen RA, Rubenstein JL: **Dlx5 regulates regional development of the branchial arches and sensory capsules**. *Development* 1999, **126**(17):3831-3846.

29. Long JE, Garel S, Depew MJ, Tobet S, Rubenstein JL: **DLX5 regulates development of peripheral and central components of the olfactory system**. *JNeurosci* 2003, **23**(2):568-578.

30. Houtmeyers R, Souopgui J, Tejpar S, Arkell R: **The ZIC gene family encodes multi-functional proteins essential for patterning and morphogenesis**. *Cellular and molecular life sciences : CMLS* 2013, **70**(20):3791-3811.

31. Kobayashi T, Kageyama R: **Expression dynamics and functions of Hes factors in development and diseases**. *Current topics in developmental biology* 2014, **110**:263-283.

32. Quint E, Zerucha T, Ekker M: **Differential expression of orthologous Dlx genes in zebrafish and mice: implications for the evolution of the Dlx homeobox gene family**. *JexpZool(MolDevEvol)* 2000, **288**:235-241.

33. Schlosser G: **Making senses: Development of vertebrate cranial placodes**. *IntRevCell MolBiol* 2010, **283C**:129-234.

34. Pandur PD, Moody SA: **Xenopus Six1 gene is expressed in neurogenic cranial placodes and maintained in differentiating lateral lines**. *MechDev* 2000, **96**:253-257.

35. Ahrens K, Schlosser G: **Tissues and signals involved in the induction of placodal Six1 expression in Xenopus laevis** *DevBiol* 2005, **288**(1):40-59.

36. Zhang C, Basta T, Jensen ED, Klymkowsky MW: **The beta-catenin/VegT-regulated early zygotic gene Xnr5 is a direct target of SOX3 regulation**. *Development* 2003, **130**(23):5609-5624.

37. Mizuseki K, Kishi M, Matsui M, Nakanishi S, Sasai Y: **Xenopus zic-related-1 and sox-2, two factors induced by chordin, have distinct activities in the initiation of neural induction**. *Development* 1998, **125**:579-587.

38. Hong CS, Saint-Jeannet JP: **The activity of Pax3 and Zic1 regulates three distinct cell fates at the neural plate border**. *MolBiolCell* 2007, **18**:2192-2202.

39. Luo T, Matsuo-Takasaki M, Thomas ML, Weeks DL, Sargent TD: **Transcription factor AP-2 is an essential and direct regulator of epidermal development in Xenopus** *DevBiol* 2002, **245**(1):136-144.

40. Suzuki A, Ueno N, Hemmati-Brivanlou A: **Xenopus msx1 mediates epidermal induction and neural inhibition by BMP4**. *Development* 1997, **124**:3037-3044.

41. Tribulo C, Aybar MJ, Nguyen VH, Mullins MC, Mayor R: **Regulation of Msx genes by a Bmp gradient is essential for neural crest specification**. *Development* 2003, **130**(26):6441-6452.

42. McLin VA, Rankin SA, Zorn AM: **Repression of Wnt/beta-catenin signaling in the anterior endoderm is essential for liver and pancreas development**. *Development* 2007, **134**(12):2207-2217.
