# Supplementary figures and images for: A gene regulatory network underlying the formation of pre-placodal ectoderm in Xenopus laevis
Source: BMC Biol. 2018 Jul 16;16:79. doi: 10.1186/s12915-018-0540-5 (PMC6048776; doi:10.1186/s12915-018-0540-5)

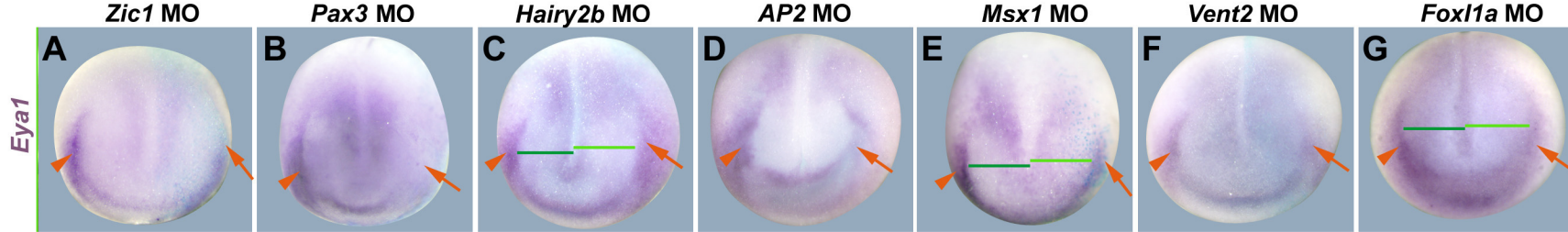

Supplement: Supplementary file 2 — Figure S1. Requirement of early ectodermal TFs for Eya1 expression in the PPE. Expression of PPE marker Eya1 in dorsal views of neural plate stage Xenopus embryos after injection of MOs blocking translation of early ectodermal TF genes. Anterior is to the bottom. Control side is shown on the left and injected side on the right (as indicated by blue LacZ staining). Reductions in the non-neural ectoderm (orange arrows) compared with the control side (orange arrowheads) are indicated. Green lines indicate broadening of the neural plate and lateral displacement of NPB markers on the injected side (bright green) versus control side (dark green). See Additional file 1: Table S3 for numbers. (PDF 254 kb) [file 12915_2018_540_MOESM2_ESM.pdf]

*Eya1*

*Zic1-GR*

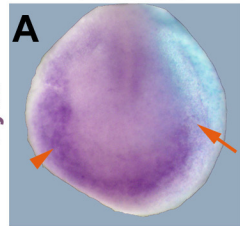

*Pax3-GR*

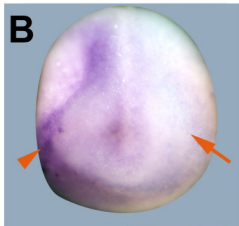

*Hairy2b-GR*

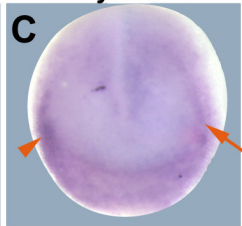

*AP2-GR*

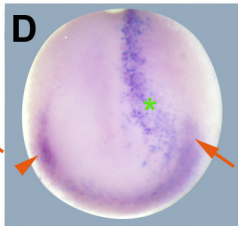

*Msx1-GR*

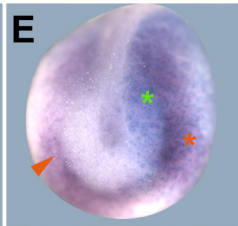

*Vent2-GR*

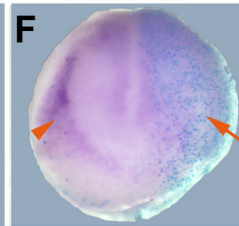

*Foxl1a-GR*

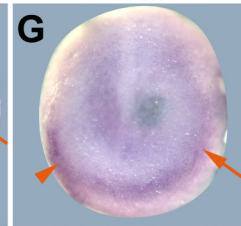

Supplement: Supplementary file 4 — Figure S3. Role of early ectodermal TFs for Eya1 expression in the PPE. Expression of PPE marker Eya1 in dorsal views of neural plate stage Xenopus embryos after injection of mRNAs for hormone-inducible early ectodermal TF genes and dexamethasone activation from stage 11–12. Anterior is to the bottom. Control side is shown on the left and injected side on the right (as indicated by blue LacZ staining). Reductions (arrows) and increased or ectopic expression domains (asterisks) in the neural (green) and non-neural ectoderm (orange) compared with the control side (arrowheads) are indicated. See Additional file 1: Tables S4 and S5 for numbers. (PDF 261 kb) [file 12915_2018_540_MOESM4_ESM.pdf]

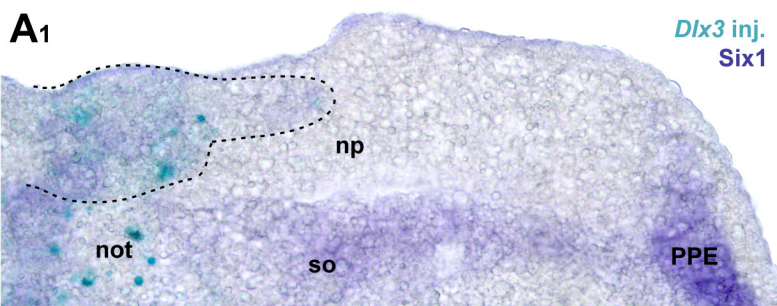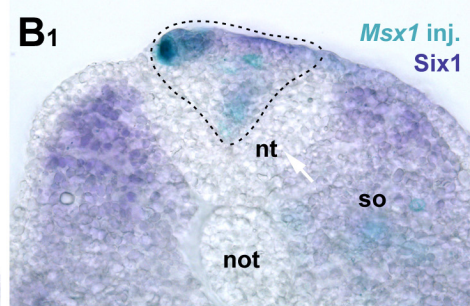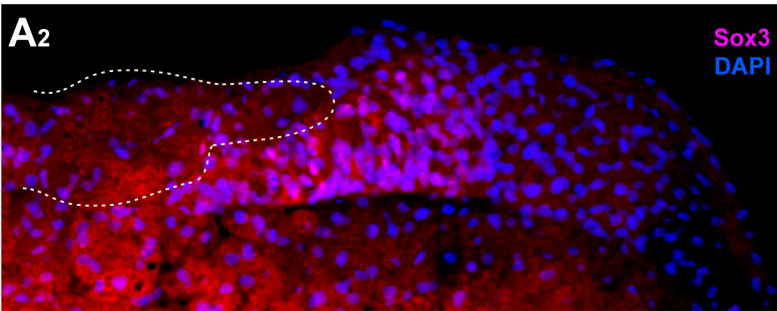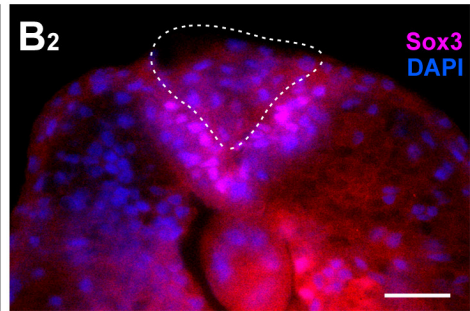

Supplement: Supplementary file 5 — Figure S4. Six1 and Sox3 expression after overexpression of Dlx3 or Msx1. Transverse sections through neural plate or neural tube of Xenopus embryos after injection of Dlx3 (A) or Msx1 (B) mRNA and in situ hybridization for Six1. Sections are shown in brightfield (A1, B1) and in an overlay of red and UV fluorescent channels (A2, B2). LacZ (turquoise in A1 and B1) reveals the extent of mRNA injection in the neural plate (hatched outlines). Nuclei are stained by DAPI (blue). Sox3 immunopositive nuclei are shown in pink. Ectopic Six1 expression is confined to Dlx3- or Msx1-injected regions of the neural plate, which lack Sox3 immunoreactivity. Abbreviations: not, notochord, np: neural plate, nt: neural tube, PPE: preplacodal ectoderm, so: somite. Bar: 50 μm (for all panels). (PDF 659 kb) [file 12915_2018_540_MOESM5_ESM.pdf]

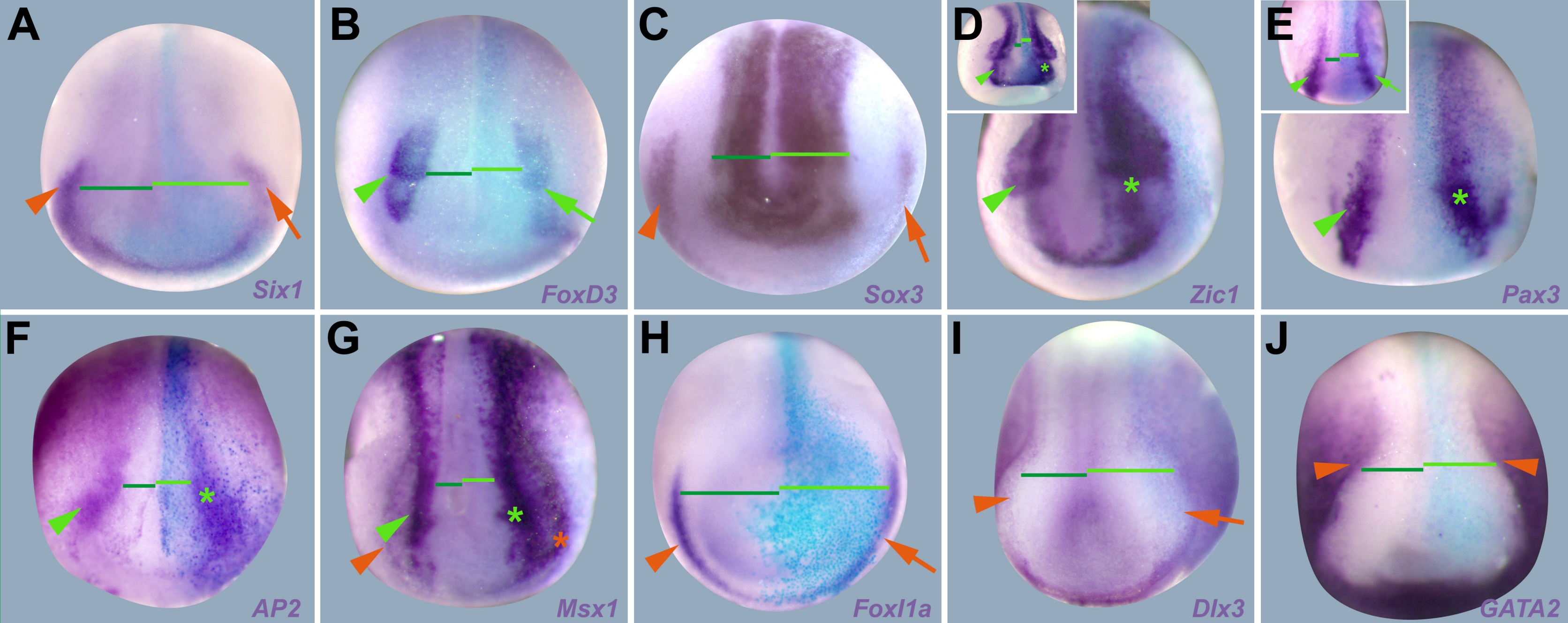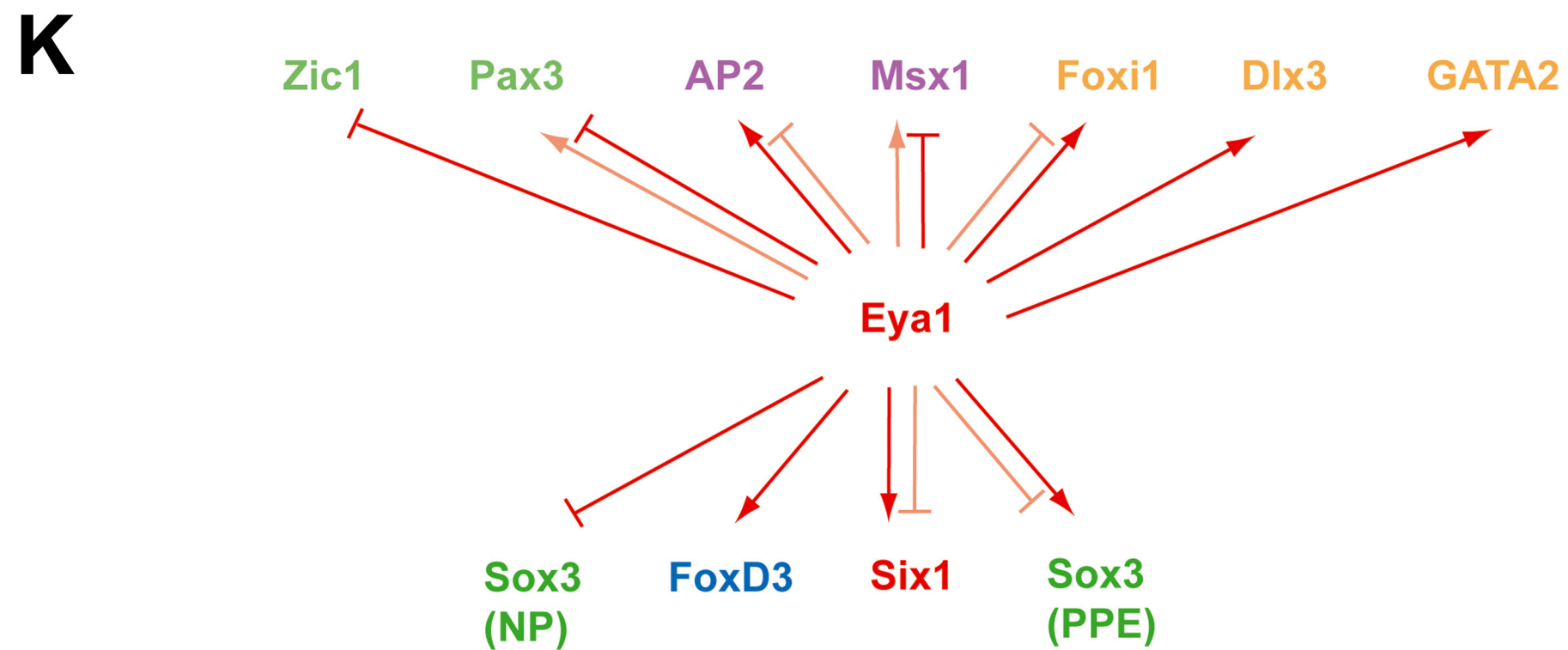

Supplement: Supplementary file 6 — Figure S5. Effects of Eya1 knockdown on NPB markers and other ectodermal TFs. A-J: Expression of PPE (Six1, Sox3), NC (FoxD3), neural plate (Sox3) markers and early ectodermal TFs in dorsal views of neural plate stage Xenopus embryos after injection of Eya1 MO1 + MO2. Anterior is to the bottom. Control side is shown on the left and injected side on the right (as indicated by blue LacZ staining). Arrowheads indicate expression domains on the control side. Reductions (arrows) and increased or ectopic expression domains (asterisks) in the neural (green) and non-neural ectoderm (orange) compared with the control side (arrowheads) are indicated. Green lines indicate broadening of the neural plate and lateral displacement of NPB markers on the injected side (bright green) versus control side (dark green). Insets show alternative phenotypes. K: Summary of regulatory interactions. Arrows indicate requirement of Eya1 for expression of TFs (reduction after Eya1 knockdown). Bars indicate requirement of Eya1 for restriction of expression of TFs (increase after Eya1 knockdown). Faint colors indicate less frequent phenotypes. See Additional file 1: Table S8 for numbers. (PDF 2013 kb) [file 12915_2018_540_MOESM6_ESM.pdf]

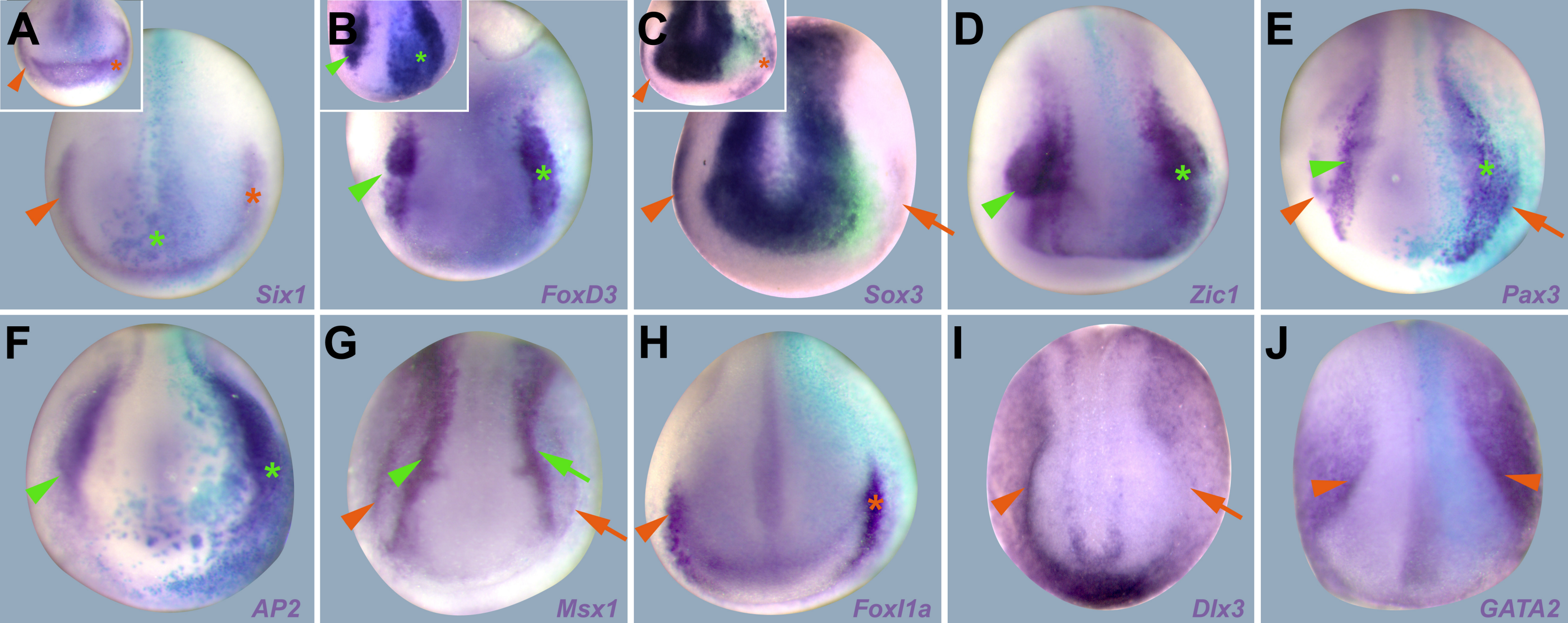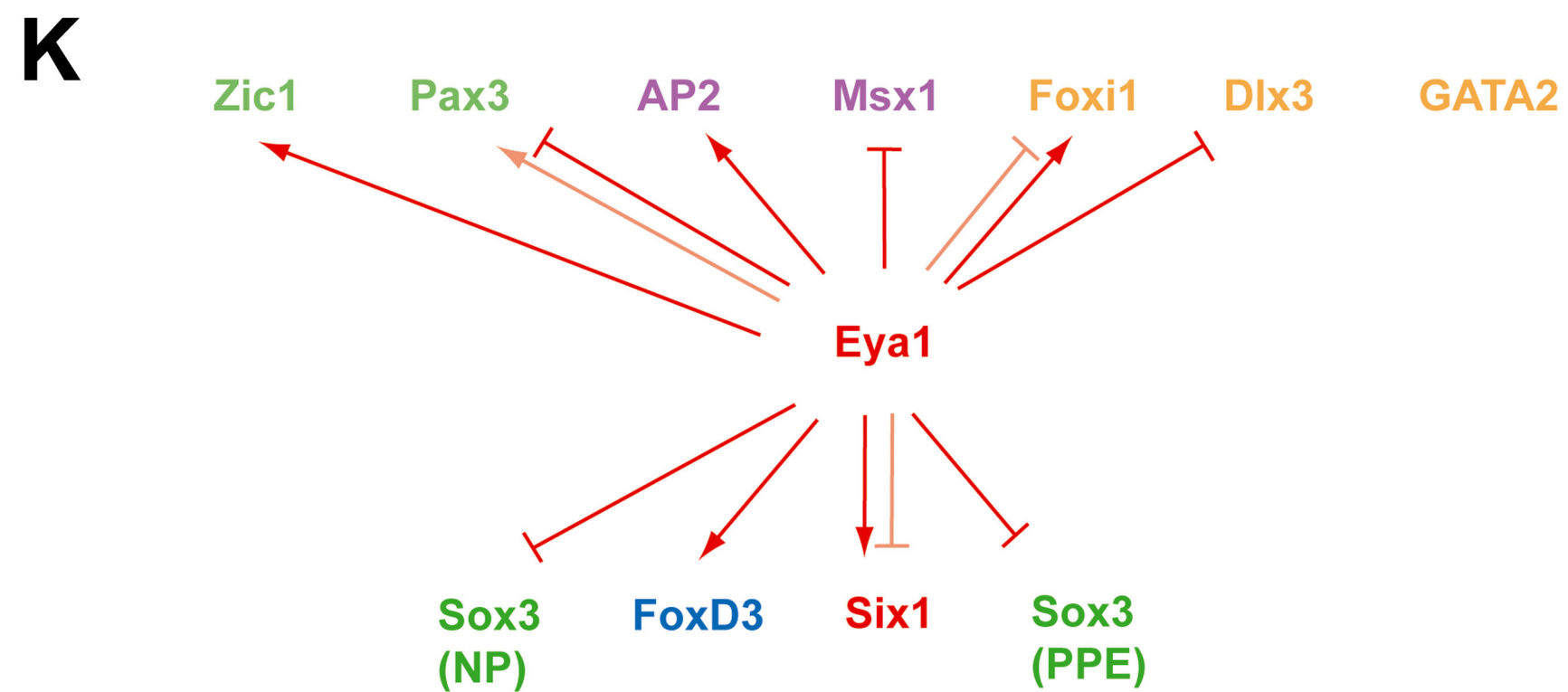

Supplement: Supplementary file 7 — Figure S6. Effects of Eya1 overexpression on NPB markers and other ectodermal TFs. A-J: Expression of PPE (Eya1, Sox3), NC (FoxD3), neural plate (Sox3) markers and early ectodermal TFs in dorsal views of neural plate stage Xenopus embryos after injection of Eya1 mRNA. Anterior is to the bottom. Control side is shown on the left and injected side on the right (as indicated by blue LacZ staining). Reductions (arrows) and increased or ectopic expression domains (asterisks) in the neural (green) and non-neural ectoderm (orange) compared with the control side (arrowheads) are indicated. Green lines indicate broadening of the neural plate and lateral displacement of NPB markers on the injected side (bright green) versus control side (dark green). Insets show alternative phenotypes. K: Summary of regulatory interactions. Arrows indicate ability of Six1 to promote expression of TFs. Bars indicate ability of Six1 to repress TFs. Faint colors indicate less frequent phenotypes. See Additional file 1: Table S9 for numbers. (PDF 1906 kb) [file 12915_2018_540_MOESM7_ESM.pdf]
