# Supplementary material for: A gene regulatory network underlying the formation of pre-placodal ectoderm in Xenopus laevis
Source: BMC Biol. 2018 Jul 16;16:79. doi: 10.1186/s12915-018-0540-5 (PMC6048776; doi:10.1186/s12915-018-0540-5)
Supplement: Supplementary file 3 — Figure S2. Cell autonomous requirements of Zic1 and Pax3 for PPE formation. Neural plates were orthotopically grafted from donor embryos to host embryos. A: Control grafts from GFP injected embryos into uninjected hosts. There is no effect on Six1 expression in the PPE (except for a slight decrease in 1/4 embryos). B: Grafting a neural plate from Pax3 MO injected embryo into uninjected hosts does not affect Six1 expression in the PPE (except for 1/10 cases). C: A neural plate graft from an uninjected embryo is unable to rescue deficits in Six1 expression in the PPE (arrow) of Pax3 MO injected embryos evident in 2/4 embryos. D: Grafting a neural plate from Zic1 MO injected embryo into uninjected hosts does not affect Six1 expression in the PPE (0/5). E: A neural plate graft from an uninjected embryo is unable to rescue deficits in Six1 expression in the PPE (arrow) of Zic1 MO injected embryos evident in 2/5 embryos. Asterisk indicates Six1 expression in graft. Arrowheads indicate the Six1 expression domain in the PPE on the control side. G: graft. (PDF 2727 kb) [file 12915_2018_540_MOESM3_ESM.pdf]

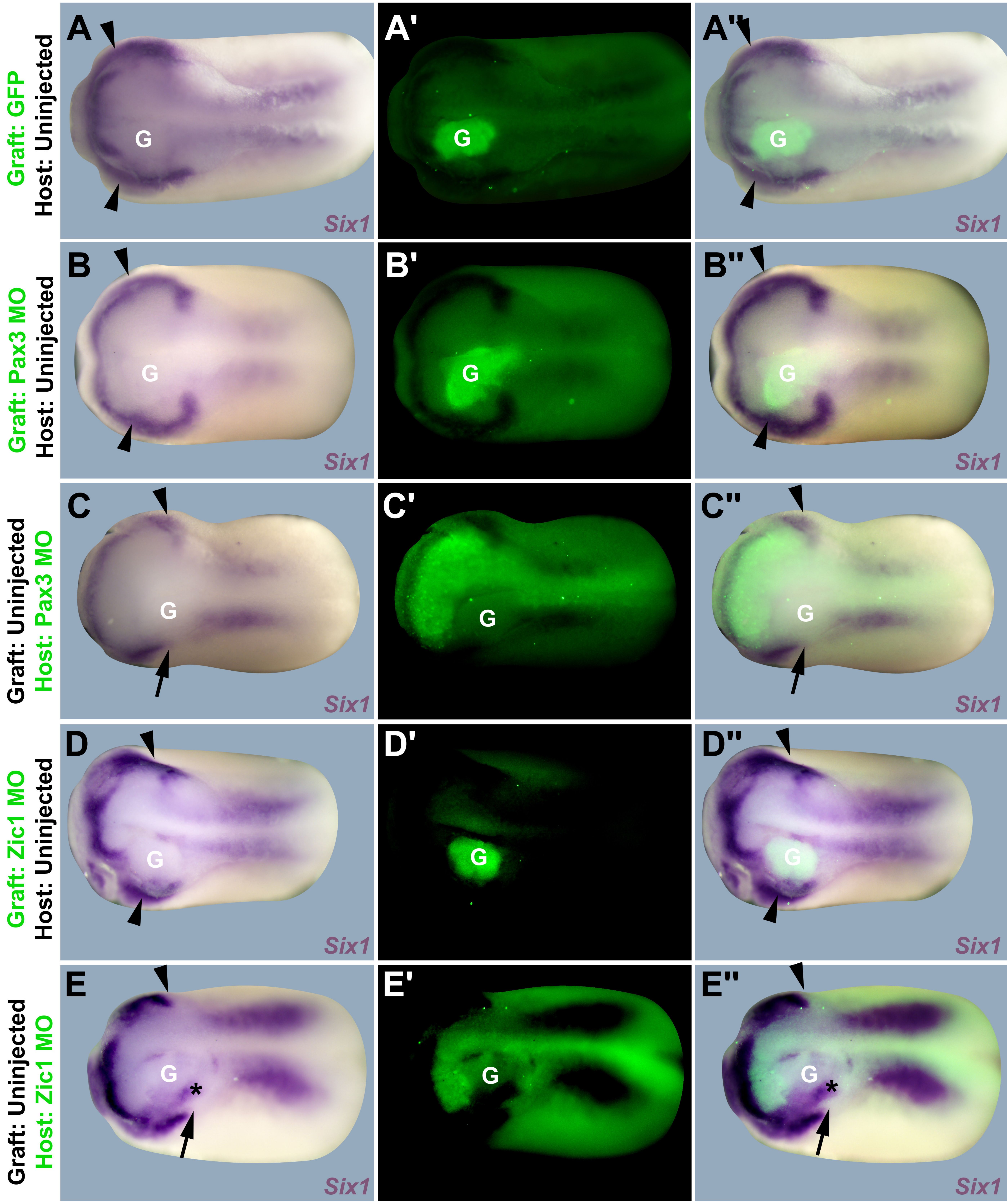

Graft: GFP
Host: Uninjected
A
G
Six1
A'
G
A''
G
Six1
Graft: Pax3 MO
Host: Uninjected
B
G
Six1
B'
G
B''
G
Six1
Graft: Uninjected
Host: Pax3 MO
C
G
Six1
C'
G
C''
G
Six1
Graft: Zic1 MO
Host: Uninjected
D
G
Six1
D'
G
D''
G
Six1
Graft: Uninjected
Host: Zic1 MO
E
G
*
Six1
E'
G
E''
G
*
Six1
