# Supplementary material for: A gene regulatory network underlying the formation of pre-placodal ectoderm in Xenopus laevis
Source: BMC Biol. 2018 Jul 16;16:79. doi: 10.1186/s12915-018-0540-5 (PMC6048776; doi:10.1186/s12915-018-0540-5)
Supplement: Supplementary file 8 — Figure S7. Models of placode specification. A: The “neural plate border state model” proposes that PPE (red) and NC (blue) are induced from a common precursor (purple) at the neural plate border, whereas the “binary competence model” proposes that they are induced from non-neural (yellow) and neural (green) ectodermal competence territories, respectively. B: In a new model that combines aspects of both these models, we propose here that there is indeed an NPB region during gastrulation, which gives rise to both PPE and NC. However, the NPB domain is not defined by a unique regulatory state but rather by the overlap of dorsally restricted neural (green) and ventrally restricted non-neural (yellow) competence factors (left panel; region of overlap: olive green). The degree of overlap decreases during gastrulation resolving into mutually exclusive non-neural and neural competence territories at the end of gastrulation (middle panel). Inducing signals from adjacent tissues induce preplacodal ectoderm (FGF, BMP-inhibitors, Wnt-inhibitors; red) and neural crest (FGF, BMP, Wnt; blue) at the border of non-neural and neural ectoderm, respectively (from [2]; modified from [70]). (PDF 2779 kb) [file 12915_2018_540_MOESM8_ESM.pdf]

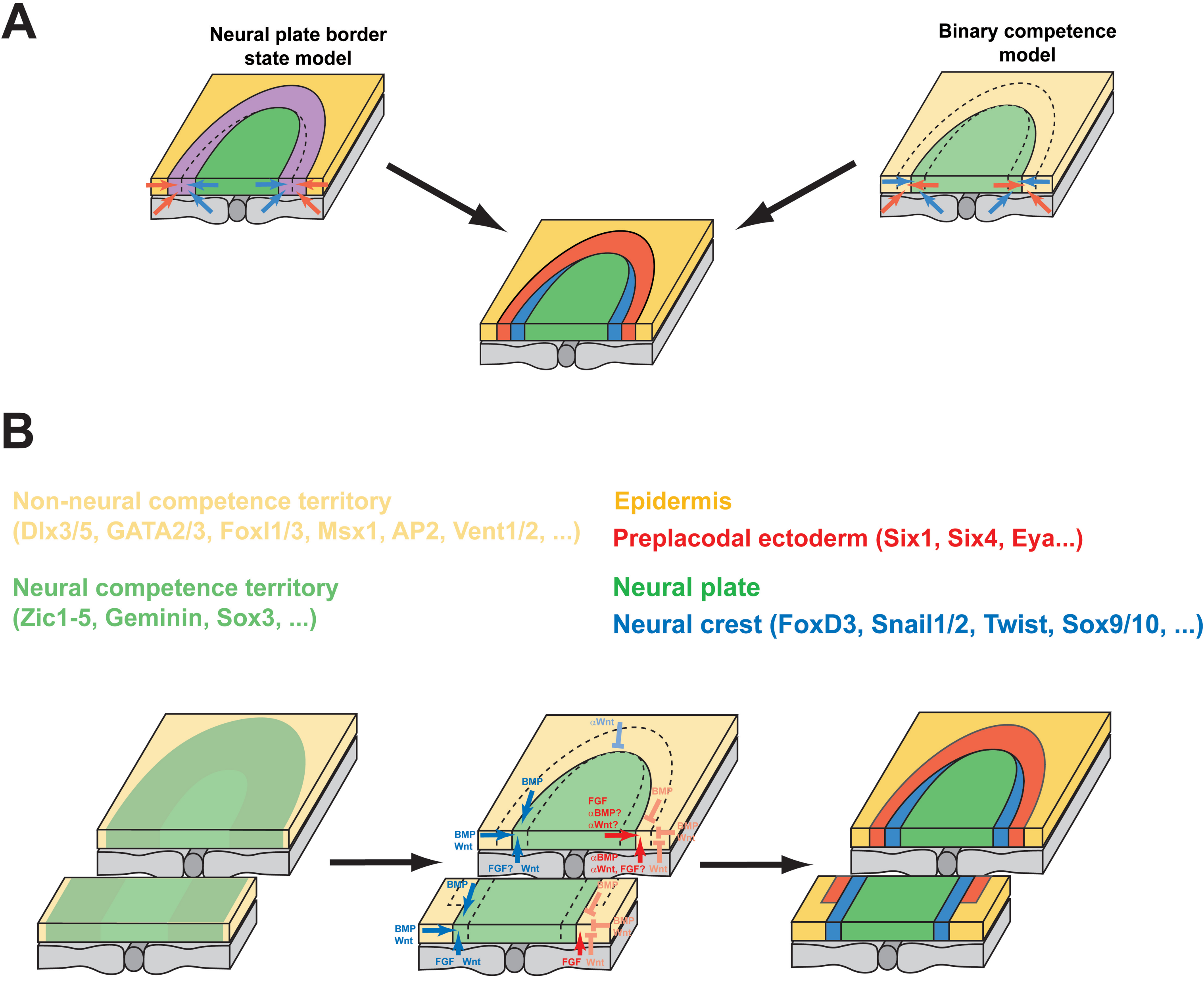
A
Neural plate border state model
Binary competence model
B
Non-neural competence territory
(Dlx3/5, GATA2/3, FoxI1/3, Msx1, AP2, Vent1/2, ...)
Neural competence territory
(Zic1-5, Geminin, Sox3, ...)
Epidermis
Preplacodal ectoderm (Six1, Six4, Eya...)
Neural plate
Neural crest (FoxD3, Snail1/2, Twist, Sox9/10, ...)
αWnt
BMP
FGF
αBMP?
αWnt?
BMP
Wnt
FGF? Wnt
αBMP
αWnt, FGF? Wnt
FGF Wnt
FGF Wnt
